# Antioxidant Activities of Natural Compounds from Caribbean Plants to Enhance Diabetic Wound Healing

**DOI:** 10.3390/antiox12051079

**Published:** 2023-05-11

**Authors:** Laura Accipe, Alisson Abadie, Remi Neviere, Sylvie Bercion

**Affiliations:** 1 UR5_3 PC2E Cardiac Pathology, Environmental Toxicity and Envenomations, Université des Antilles, BP 250, CEDEX, 97157 Pointe à Pitre, France; 2 CHU Martinique, University Hospital of Martinique, 97200 Fort de France, France

**Keywords:** wound healing, diabetes, reactive oxygen species, inflammation, plants

## Abstract

Diabetic wound healing is a global medical challenge. Several studies showed that delayed healing in diabetic patients is multifactorial. Nevertheless, there is evidence that excessive production of ROS and impaired ROS detoxification in diabetes are the main cause of chronic wounds. Indeed, increased ROS promotes the expression and activity of metalloproteinase, resulting in a high proteolytic state in the wound with significant destruction of the extracellular matrix, which leads to a stop in the repair process. In addition, ROS accumulation increases NLRP3 inflammasome activation and macrophage hyperpolarization in the M1 pro-inflammatory phenotype. Oxidative stress increases the activation of NETosis. This leads to an elevated pro-inflammatory state in the wound and prevents the resolution of inflammation, an essential step for wound healing. The use of medicinal plants and natural compounds can improve diabetic wound healing by directly targeting oxidative stress and the transcription factor Nrf2 involved in the antioxidant response or the mechanisms impacted by the elevation of ROS such as NLRP3 inflammasome, the polarization of macrophages, and expression or activation of metalloproteinases. This study of the diabetic pro-healing activity of nine plants found in the Caribbean highlights, more particularly, the role of five polyphenolic compounds. At the end of this review, research perspectives are presented.

## 1. Introduction

Diabetes is one of the most prevalent diseases in the world, affecting nearly 537 million adults in 2021, according to the International Diabetes Federation [1]. This figure is expected to reach 780 million in 2045. Chronic hyperglycemia leads to many complications, including failure to heal. Many diabetic patients, about 30%, will develop foot ulcers following trauma [2]. These are chronic wounds that do not heal and can lead to amputations.

Numerous cellular and molecular studies have been conducted to elucidate this healing defect in diabetic patients. It seems obvious that this delay is multifactorial. The main causes that have been identified are as follows: imbalance in the expression of cytokines and growth factors, increased metalloproteinase activity, increased oxidative stress, and increased formation of glycation end products (AGEs). In addition, impaired neo-angiogenesis, dysfunction of cells involved in the wound healing process, and a chronic inflammatory state have also been cited as responsible for delayed wound healing [3,4,5,6,7,8]. The chronic inflammatory state is associated with different factors: a defect in macrophage polarization [9,10,11], the sustained activity of the NOD-like receptor family, the pyrin domain containing 3 (NLRP3) inflammasome in the wound [12], and the exacerbated NETosis phenomenon [5,13,14].

In addition, the involvement of reactive oxygen species (ROS) during wound healing has been studied. Hydrogen peroxide (H_2_O_2_) and superoxide (O_2_^•−^) are important intracellular secondary messengers that regulate different phases of wound healing, including cell recruitment, production of cytokines and factors involved, cell migration and proliferation, and angiogenesis [15,16,17,18]. Prolonged hyperglycemia in diabetic patients leads to an increase in the production of ROS, because it favors the consumption of oxygen by the mitochondria, which in the long term damages the functions of the mitochondria and leads to overactivity of the NADPH oxidase (NOX), which are ROS-producing enzymes [19]. In addition, hyperglycemia also induces an increase in the amount of oxidative stress via the polyol pathway or protein kinase C (PKC) signaling [19,20], and via the accumulation of terminal glycation products (AGEs) [21,22]. The involvement of ROS in the failure of healing in diabetic patients is complex. Numerous studies have attempted to highlight the role of ROS in delayed wound healing: the impairment of redox homeostasis due to increased production of ROS induces oxidative damage at the cellular level, which inhibits wound healing [23,24]. Moreover, the Nuclear factor (erythroid-derived 2)-like 2 (Nrf2) signaling pathway, involved in the transcription of antioxidant genes, is also impacted in diabetes [25]. It is, therefore, oxidative stress, which represents an increase in the level of ROS and/or a decrease in antioxidant defenses [22], which is involved when the healing defect [26]. Indeed, this oxidative stress modifies many processes, in particular, the modification of the functioning of macrophages and neutrophils [27,28,29], adverse effects on the migration and proliferation of endothelial cells, fibroblasts, and keratinocytes [17,30], and modification of the expression and activity of metalloproteinases [31,32]. In addition, collagen production is impaired [33], and the extracellular matrix is altered [34]. All these changes are involved in stopping the healing process in diabetic patients.

The use of different plant extracts with antioxidant properties is known to improve wound healing [35].

This review will focus on evaluating the effect of oxidative stress on different mechanisms altered in diabetic wound healing and the use of Caribbean plants as therapeutic leads.

## 2. Effect of Oxidative Stress on Different Mechanisms Altered in Diabetic Wounds

### 2.1. Oxidative Stress and MMPs

Matrix metalloproteinases (MMPs) are proteases or proteolytic enzymes that are involved in many cellular processes. One of the characteristics of chronic wounds is a high proteolytic state. Various studies have shown that MMP9 is involved in delayed wound healing [36]. Indeed, MMP9 is the metalloproteinase whose expression is most increased in chronic wounds, and it induces apoptosis of keratinocytes in diabetic wounds [37,38]. Various studies have identified the involvement of ROS in the activity and expression of MMPs. The review by Nelson K et al., 2004, assessed the effect of ROS on the expression and activation of MMPs: ROS are fine regulators [39,40]. Indeed, H_2_O_2_ can, for example, modulate the expression of MMPs via Mitogen-activated protein kinase (MAPK), but also improve the activity of MMP-2 and MMP-9 [32]. In addition, hyperglycemia has been shown to increase MMP-9 expression via ROS-induced MAPK signaling in brain astrocytes [41]. Oxidative stress has also been implicated in epigenetic modification of the MMP-9 promoter linked to the development of diabetic retinopathy [42]. Increased ROS in diabetic patients may therefore be responsible for the increased expression of MMP-9 and the elevated proteolytic state at the wound site. Interestingly, the use of eugenol, an antioxidant molecule, particularly on hydrogen peroxides, decreased the activity and expression of MMP-9 via the inactivation of extracellular signal-regulated kinases (ERK) [43]. However, the use of a specific inhibitor of MMP 9 accelerated the healing of diabetic mice [44]. It, therefore, appears possible that modulation of ROS could inhibit the overexpression of MMP9 in diabetic wounds and/or inhibit MMP9 activity.

### 2.2. Oxidative Stress and AGEs

The hyperglycemia found in diabetic patients favors the glycation of proteins. This is a non-enzymatic reaction that occurs between a carbohydrate and a molecule with a free amine group, such as proteins. It is a spontaneous, irreversible, and cumulative reaction called the Maillard reaction. The end products of glycation or AGEs are, therefore, a group of structurally heterogeneous molecules that result from a long and complex process. Hyperglycemia and constant oxidative stress lead to an increase in the formation of these AGEs via a phenomenon called glycoxidation, i.e., oxidation and glycation caused by the formation of dicarbonyls (methylglyoxal and glyoxal) [45]. During diabetic wound healing, AGEs found in the dermis of patients form cross-links with collagen, which reduces the ability of keratinocytes to migrate [46,47]. Moreover, the signaling induced after binding of AGEs to their RAGE receptor leads to an increase in ROS production via NADPH oxidase [48,49], an increase in inflammation via the transcription factor nuclear factor-kappa B (NF-κB) [50,51,52], and an increase in apoptosis of fibroblasts and keratinocytes [38,53].

Moreover, the increase in oxidative stress accelerates the formation of AGEs [54], and the increase in AGEs increases the formation of ROS: this positive feedback loop, therefore, leads to an increase in oxidative stress and an increase in these harmful effects in the wound bed of diabetic patients, which leads to the inhibition of the repair process.

### 2.3. Oxidative Stress and Inflammation

#### 2.3.1. Macrophage Polarization

It is clear that macrophage plasticity is a key factor during the inflammatory phase. Appropriate polarization allows the repair process to proceed smoothly [10]. Indeed, macrophages trigger both the onset of the inflammatory phase and its resolution. Classically, the M1 subpopulation or proinflammatory macrophages are involved in bactericidal activities and, thus, in the onset of the inflammatory phase. In comparison, the M2 or anti-inflammatory subpopulation is involved in the resolution of the inflammation [55,56,57].

Physiologically, ROS are essential in the induction and maintenance of M1 polarization via the NF-κB and MAPk signaling pathways, which induce the transcription of pro-inflammatory genes. Different mechanisms are proposed and would involve NADPH oxidase and H_2_O_2_ production [56]. In addition, ROS are also involved in M2 polarization. Zhang et al. have shown that inhibition of NOX-derived superoxide production inhibits M2 polarization and that ROS are involved in the late phase of polarization via ERF signaling [58]. Many parameters influence the role of ROS during polarization, including the stage of differentiation and the redox state of the cells. In diabetic pathology, there is a stable intrinsic change in the hematopoietic cells: macrophages are hyperreactive and show a hyperpolarization of the M1 type [7,59]. Moreover, M2 macrophages in diabetic patients are of the M2b type: there is a deficiency in the transition from the inflammatory phenotype characterized by M1 and M2b to a reparative phenotype M2a and M2c [11]. Under hyperglycemic conditions, it has been shown in RAW264.7 that ROS increase the transcription of mRNAs of pro-inflammatory cytokines such as the induced form of nitric oxide synthase (iNOS), interleukin-6 (IL-6), or TNF-α and diminish the transcription of mRNAs of the M2 markers, macrophage mannose receptor (CD206), and arginase-1 (Arg-1) [60]. Redox modifications and ROS production are involved in macrophage plasticity and regulate their polarization [61,62]. The increase in oxidative stress could therefore be a cause of the polarization defect of macrophages during the healing of diabetic foot wounds.

#### 2.3.2. NLRP3 Inflammasome

Inflammasomes are cytosolic protein complexes that are formed to enable immune responses to infection and cell damage. Activation of an inflammasome triggers the activation of caspase 1, and the re-release of IL-1β, a pro-inflammatory mediator, and IL-18, which induces the expression of INF-γ and mediates the cytolytic activity of natural killer cells and T cells [63]. Many mechanisms of activation have been proposed in view of the number of known stimuli. ROS probably contribute to the activation of the NLRP3 inflammasome, which is supported by the ability of a large number of activators to trigger mitochondrial ROS production [64]. This hypothesis emerged when inhibition of NOX-derived ROS prevents caspase 1 activation and IL-1β release [65]. Surprisingly, human peripheral blood mononuclear cells lacking NOX activity show normal NLRP3 inflammasome activity [63]. Nevertheless, it can be assumed that another source of ROS, e.g., xanthine oxidoreductase, could produce a sufficient amount of ROS for activation.

In diabetic patients, it has been found that NLRP3 expression and activity are strongly increased [66,67]. Xanthine oxidoreductase activity is elevated in chronic wounds, which leads to an overproduction of ROS [68]. It can therefore be assumed that the increase in ROS derived from NOX or xanthine oxidoreductase in diabetic patients increases the activity of the NLRP3 inflammasome in macrophages, which impairs wound healing. This hypothesis is in agreement with the study of Mirza et al., who observed that high inflammasome activation in macrophages is associated with increased interleukin 1 β (IL-1β), which impairs wound healing in diabetic subjects [12].

#### 2.3.3. NETosis

NETosis is a cellular mechanism in which neutrophils release extracellular neutrophil traps (NET). These are DNA-like genetic materials that are released with granular antimicrobial proteins. It is, therefore, decondensed chromatin with several associated proteins, such as histones that possess intrinsic antimicrobial activity and proteins with bactericidal activity, such as neutrophil elastase, myeloperoxidase (MPO), gelatinase, and other proteins, which destroy virulence factors [69,70]. After recognition of a stimulus, neutrophils are activated: NADPH oxidase is then activated via protein kinase C- MAPK. Numerous enzymes are then activated, including protein-arginine deiminase type 4 (PAD4), which leads to the citrullination of histones resulting in chromatin decondensation. The plasma membrane is perforated, which allows the release of NETs [70]. Diabetic neutrophils produce more superoxide [71] and are more sensitive than healthy neutrophils to NETosis [5]. The increase in NETosis found in diabetic patients is a factor that would be responsible for the delay in wound healing. Indeed, neutrophil depletion in diabetic mice promotes wound healing [72]. Neutrophils cultured in a high glucose environment show an overproduction of ROS and a high rate of NET production. The use of two NADPH oxidase inhibitors, apocynin and diphenyleneiodonium chloride, decreases the formation of NETs in high-glucose neutrophils and in patients with diabetes: the formation induced by high glucose is dependent on NADPH oxidase [73]. The use of molecules capable of inhibiting NETose formation, such as hydrogen sulfide, improves diabetic wound healing [74]. In addition, NETs have been shown to promote the release of IL-1β by macrophages, which promotes inflammation in these wounds [75]. This increase in IL-1β can be explained by the ability of NETs to activate the NLRP3 inflammasome [76]. Neutrophils also play a role in macrophage polarization after NETs formation: indeed, in the presence of NETs, M2 macrophages secrete proinflammatory cytokines, and M1s go into apoptosis via PAD4 and release DNA material which in turn can play a detrimental role on surrounding cells [77].

NETosis increases inflammation during diabetic wound healing. Decrease NETosis in diabetic patients could therefore be a pharmacological lever to promote their wound healing.

However, in the pathology of diabetes, we find an overactivation of NADPH oxidase [19]. It can therefore be assumed that the increase in oxidative stress in diabetic patients favors the NETosis phenomenon, which inhibits healing.

### 2.4. Oxidative Stress and Nrf2

The transcription factor Nrf2 or Nuclear factor erythroid 2-related factor 2 is the primary regulator of oxidative stress. Indeed, it protects cells against oxidative damage [78]. The main role of this transcription factor is to detect the accumulation of ROS and to activate the transcription of the antioxidant defense system [79]. In general, Nrf2 is linked to its inhibitor Kelch ECH associating protein 1 (Keap1). When oxidative stress increases, Keap 1 is modified, allowing Nrf2 proteins to be translocated into the nucleus and bind to target genes with an antioxidant response element (ARE) such as NADPH quinone oxidoreductase, heme oxygenase 1 (HO-1), or glutathione S transferases [80]. HO-1 is an enzyme that degrades heme and generates antioxidant molecules, biliverine and carbon monoxide (CO), and iron ions that regulate important processes, including inflammation, apoptosis, cell proliferation, and angiogenesis [81]. During wound healing, Nrf2 decreases oxidative stress in cells and plays a role in epithelial cell proliferation, migration, and apoptosis [78,79,82]. In addition, Nrf2 regulates the level of MMP9 [83].

The activity of Nrf2 in diabetic patients is altered. Indeed, at the beginning of the healing process, Nrf2 activity leads to an increase in the transcription and activity of antioxidant enzymes. Nrf2 activity decreases rapidly, resulting in a failure to compensate for ROS production by antioxidant enzymes [83]. This defect is explained by an inhibition of the nuclear translocation of Nrf2 under conditions of chronic hyperglycemia. This nuclear translocation defect is the result of the sequestration of Nrf2 by its inhibitor Keap1 in the cytoplasm [84]. Indeed, Rabbani et al. used siRNA therapy against Keap1 in a mouse model of diabetic wound healing. Decreased expression of Keap1 restored the antioxidant function of Nrf2 and improved wound healing [85]. Furthermore, it was shown in diabetic mice that HO-1 expression was impaired, which correlates with a defect in neovascularization. The delivery of HO-1 using adenoviral vectors was able to accelerate wound healing in these mice [86]. The decrease in HO-1 expression can be explained by the lack of activity of the transcription factor Nrf2. In dermal fibroblasts from diabetic rats, there is an increase in superoxide formed by NOX, a decrease in antioxidant capacity, and a decrease in Nrf2 expression [87], which could also explain the delayed wound healing in patients.

The modulation of oxidative stress in diabetic patients, therefore, seems to be an important therapeutic avenue to promote healing. Indeed, the increase in oxidative stress could be responsible for the increase in the activity of metalloproteinases and, more particularly, MMP9 in diabetic foot wounds, but also for the accumulation of glycation end products and their harmful effects via its receptor RAGE. Furthermore, elevated ROS levels may also be responsible for the persistent pro-inflammatory state within wounds by maintaining M1 polarization of macrophages and increasing NLRP3 inflammasome activation in these cells. In addition, hyperglycemia and oxidative stress inhibit the transcription factor Nrf2, which can no longer promote antioxidant enzymes. Diabetic wounds are therefore maintained in a pro-oxidative and pro-inflammatory state, which leads to the arrest of the healing process.

## 3. Improvement in Wound Healing by Plant Extracts

**Figure 1:**
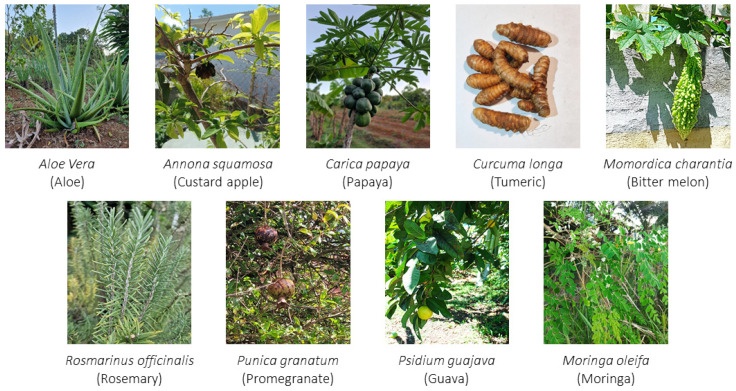
Photographies of selected plants improving the healing of diabetic wounds.

In the literature, numerous reviews report the use of plants to improve wound healing [88,89,90] and diabetic wounds [91,92,93], but data from ethnopharmacological surveys on the traditional use of plants specifically to treat diabetic foot wounds are still rare. However, it is information on efficacy recognized by users that is generally the source of scientific work at the origin of research carried out in vitro on diabetic animals. This is the case, for example, of the work carried out by Nayak et al., following information given to these scientists by patients attending the Diabetic Wound Clinic in Trinidad [94]. A search for plants in the Caribbean in the book by Jacques Fournet [95], which are known to improve healing in vitro in diabetic situations, revealed nine plants (Figure 1, Table 1) that contain phenolic compounds that are known to be antioxidants. In addition, they can also modulate the expression and activity of MMPs and inhibit the formation of glycation end products. The extracts of these plants can also have anti-inflammatory properties either by modifying the polarization of macrophages or by inhibiting the activation of the NLRP3 inflammasome. Moreover, they can also decrease oxidative stress by modulating the expression or activity of Nrf2.

### 3.1. Selected Plants

#### 3.1.1. *Aloe vera* (L.) Burm.f., 1768

Aloe Vera is a plant of the Liliaceae family. It is a rosette plant of about 80 cm tall with shallow roots with a null or short stem, bearing long green leaves and small light yellow spines [95]. Used as a medicinal plant in many cultures, it is known to treat bruises and wounds. Chithra et al. were able to evaluate the positive effect of the freeze-dried gel of crushed mature leaves on the wound healing of diabetic rats. The gel was able to improve collagen deposition and granulation tissue [96]. Furthermore, aloe gel is known to decrease the activity and expression of MMP9 in LPS-activated Peripheral Blood Mononuclear Cells (PBMCs) [121]. Aloe extracts also possess anti-glycative activity: the anthraquinone present in the methanolic extract inhibits the formation of AGEs and CML in a BSA/glucose model [122]. Moreover, aloe is known for its anti-inflammatory properties. Indeed, aloe gel encapsulated in chitosan-based nanohydrogel modulates macrophage polarization in rats during wound healing: it leads to a decrease in M1 macrophages after 3 days and an increase in M2 after 14 days [123]. Aloe can also decrease IL-1β secretion and the expression of NLRP3 components by inhibiting NF-κB, p38, c-Jun N-terminal kinase (JNK), and ERF in THP-1 macrophages [124]. In a mouse model of acute colitis, polysaccharides isolated from aloe vera gel were able to increase Nrf2 expression [125]. The different activities of A. vera extracts may explain the beneficial effect found in the rat model of diabetic scarring.

#### 3.1.2. *Annona squamosa* L., 1753

The custard apple or sugar apple is a shrub of about 1.5–6 m, often tortuous, of the Annonaceae family, with greenish-yellow lanceolate leaves of 2–3 cm and a bumpy green fruit composed of multiple segments welded together. Locally it is used for superficial skin disorders and stimulates digestion. The crushed seeds are used as an insecticide [95]. Ponsaru et al. have demonstrated the beneficial effect of the dried ethanolic extract of young leaves on the healing of diabetic rats. Indeed, the extract decreases the time of re-epithelialization, increases wound contraction, and improves collagen deposition [98]. In addition, custard apple leaf extract decreases the level of MMP1 in rats after exposure to ultraviolet B (UVB) [126]. It thus appears that cinnamon apples can modulate the activity of MMPs and thus improve wound healing by this means.

#### 3.1.3. *Carica papaya* L., 1753

The papaya or pawpaw is a shrub of the Caricaceae family about 3–7 m tall with a hollow trunk, which has a greenish or grayish bark marked with horizontal leaf scars. Its leaves are palmate and gather at the top of the trunk with a long petiole. Locally it is used as a vermifuge, a digestive stimulant, and soothes skin disorders and ulcerated wounds [95]. Its beneficial effect on wounds was confirmed by Nayak et al., who used unripe fruit juice on wounds of diabetic rats. This extract was able to decrease the time of re-epithelialization and increase the contraction of the wound [94]. The dried ethanolic extract of dried leaves containing caffeic acid and rutin decreased the expression of MMP1 and MMP3 in Normal Human Dermal Fibroblasts (NHDFs) irradiated with UVB [127]. In addition, papaya juice extract inhibits enzymes of the polyol pathway, which are involved in the formation of glycation end products: papaya could therefore exert an anti-glycative effect [128]. It may also reduce inflammation caused by macrophages. Indeed, the aqueous extract of papaya leaves modulates the expression of surface markers of macrophages associated with tumors isolated from Dalton’s lymphoma mice.

#### 3.1.4. *Curcuma longa* L., 1753

Turmeric is a large aromatic tuberous bright yellow fleshy rhizome of the Zingiberaceae family of about 0.45–1 m, having large lanceolate sword-shaped leaves with a pale-yellow sheathing base. Locally it is used for skin and liver disorders and as a hypocholesterolemic agent [95]. Sidhu et al. evaluated the effect of an aqueous extract on the wounds of diabetic rats. This extract increased epithelial migration and improved re-epithelialization [104]. Curcumin and other curcuminoids (demethoxy and bisdemethoxy-curcumin) inhibit MMP1 expression in HaCaT keratinocytes and human fibroblasts after UVB exposure [129]. In addition, treatment with curcumin in diabetic rats decreased the cross-linking between AGEs and collagen [130]. The anti-inflammatory activity of turmeric was also evaluated: first, a gel of turmeric was applied to the wounds of mice. The gel induced an increase in ARG-1, which is a marker of M2 polarization, and a decrease in iNOS, which is a marker of M1 [131]. This same observation could be made on RAW264.7 cells [132]. In a second step, curcumin was able to inhibit stress-induced NLRP3 inflammasome activation, which is accompanied by decreased IL-1β production in rats with mild stress [133]. In addition, turmeric extracts can enhance the activation of antioxidant enzymes by activating the transcription factor Nrf2 in lymphoma ascites cells [134].

#### 3.1.5. *Momordica charantia* L., 1753

Bitter melon, or pawoka, is a slender climbing vine of the Cucurbitaceae family with rigid stems that can reach up to 8 m, with 10 to 15 cm long leaves cut into irregularly shaped lobes. The flowers are yellow and cornet-shaped, with long stalks giving off a vanilla odor [95]. It is traditionally used to treat various diseases such as stomachaches, colds, fever, gout, rheumatism, and wounds. Hussan et al. were able to improve the wound healing of diabetic rats using an aqueous extract of the fruit. This extract was able to improve re-epithelialization and protein content and increase TGF-β1 expression [109]. In addition, the dried ethanolic leaf extract is known to decrease the expression of MMP9 and MMP2 in PLS10 cells [135]. Aljohi et al. were able to evaluate the antiglycative properties of a methanolic extract of the pulp. They were able to obtain an inhibition of the formation of methylglyoxal-derived AGEs and CMLs [136]. *M. charantia* can also modulate macrophage polarization in mice. Indeed, the addition of pawoka powder to the diet of mice on a high-fat diet induces a decrease in M1 macrophages that is accompanied by an increase in M2 macrophages. The acetone fraction of fruit juice can also decrease NLRP3 expression in RAW264.7 [137], which is in agreement with the observations of Nerurkar et al. in mice [138]. In addition, pawoka polysaccharides improve antioxidant capacity by decreasing the level of malondialdehyde and increasing the expression of antioxidant enzymes such as superoxide dismutase and glutathione via enhancing the entry of Nrf2 into the nuclei of hypothalamus cells in a D-galactose induced aging rat model [139].

#### 3.1.6. *Moringa oleifera* Lam., 1785

Moringa is a small tree of the Moringaceae family about 10 m tall with a greyish-white, cracked bark, with spreading and fragile branches where we find feathery-looking three-pinnate leaves and very fragrant flowers arranged in clusters of five unequal yellowish-white petals [95]. It is commonly used for its medicinal properties: antimicrobial, antidiabetic, and anti-cancer. Al-Ghanayem et al. reported the healing activity of methanolic extract of leaves on diabetic rats. This extract increases re-epithelialization and improves wound contraction, collagen deposition, and angiogenesis [110]. In addition, iso-thiocyanates present in moringa seeds decreased the expression of MMP1, MMP3, and MMP9 in HaCaT cells [140]. The dried methanolic extract and the aqueous extract of the dried leaves inhibited the formation of terminal glycation products more than the well-known inhibitor aminoguanidine [141]. In mouse spleens, the administration of moringa leaf extract decreases the expression of NLRP3, which is accompanied by an increase in the expression of antioxidant enzymes after methotrexate-induced stress [142]. The increase in enzyme expression may be due to an action on the Nrf2 factor. Indeed, in the liver tissue of a mouse model of steatosis, the aqueous extract of the leaves increases the expression of Nrf2 and glutathione [143].

#### 3.1.7. *Psidium guajava* L., 1753

The guava is a fairly twisted fruiting shrub about 2–10 m tall with a smooth, thin green or reddish bark that peels off in patches. It has extremely hard wood and very divided branches with oblong, obtuse, or acute leaves with prominent veins covered with a fine down on their inner side. The flowers are white, and the buds are used in the Caribbean as an infusion against diarrhea [95]. The dried acetone extract from the leaves improves the healing of diabetic rats, according to the study of Kumari et al. [115]. In DU-145 prostate carcinoma cells, an aqueous extract of guava leaves decreased the expression of MMP2 and MMP9, accompanied by an increase in TIMP2 [144]. In addition, the aqueous extract of dried leaves inhibited the formation of AGEs in the BSA/glucose system [145]. The anti-inflammatory activity of flavonoids extracted from leaves could be evaluated. Indeed, in the mouse model of chronic pancreatitis, the administration of flavonoid extract decreased the expression of NLRP3 and caspase 1, accompanied by a decrease in IL-1β and IL18 [146].

#### 3.1.8. *Punica granatum* L., 1753

Grenadier is a thorny shrub about 1–4 m tall with a twisted trunk and a grayish bark. It has simple opposite and lanceolate light green leaves and a spherical fruit up to 10 m in diameter forming a crown at the top, and is covered with a hard and thick red bark. It is used in the Caribbean for sore throat and diarrhea [95]. Yan et al. evaluated the effect of ethanolic extract from the dried skin of the fruit on diabetic rats; this extract improves re-epithelialization, collagen deposition, angiogenesis, and expression of factors such as TGF-β1, vascular endothelial growth factor (VEGF), and epidermal growth factor (EGF) [117]. The effect of pomegranate on the activity and expression of MMPs was evaluated. Indeed, the aqueous extract of pomegranate skins inhibited the activity of MMP2 and MMP9 in lung tissue as well as their expression in a mouse model of LPS-induced lung inflammation [147]. Similarly, the polyphenols present in pomegranate juice inhibit the formation of terminal glycation products of glyceraldehyde-derived AGEs [148]. Pomegranate juice, as well as punicalagin, one of its constituents, improved M2 polarization in J774A.1 macrophages, with an increase in the secretion of IL-10 anti-inflammatory cytokines and a decrease in the expression of IL-6 pro-inflammatory cytokines [149]. Moreover, the known antioxidant activity of pomegranate can be explained by its ability to activate Nrf2 translocation in the nucleus and inhibit NF-κB in SH-SY5Y neuroblastoma cells. In addition, the fruit wine extract was also able to increase the activity of antioxidant enzymes: superoxide dismutase and glutathione peroxidase [150].

#### 3.1.9. *Rosmarinus officinalis* L., 1753

Rosemary is a green shrub of about 0.5–2 m in height and is very branched and leafy. It has a woody stem covered with a greyish bark dividing into many opposite branches; its leaves are sessile opposite and curled at the edges. It is used in cooking as an aromatic [95], but it is also used traditionally for its antispasmodic, anti-aging, respiratory, and digestive properties. Abu-Al-Basal et al. evaluated the effect of an aqueous extract on wound healing in diabetic mice. The extract improved wound contraction and angiogenesis [119]. Furthermore, in the BSA/fructose system, rosemary leaf extract inhibited the formation of AGEs [151]. The different components of rosemary are known to modulate the NLRP3 inflammasome. Indeed, carnosic acid specifically inhibits NLRP3 activation in human and murine macrophages by suppressing mitochondrial ROS production [152]. Rosemary extract decreases MMP1 expression in fibroblasts and 3D reconstructed skin models [153]. Rosemary-derived carnosol is able to activate Nrf2 in HCT116 cells, inducing activation of sestrin 2, which combats oxidative stress and DNA damage [154].

As previously described, the different Caribbean plants can act on the different mechanisms that impact the healing of diabetic foot wounds. The different effects have been listed in Table 2, Table 3, Table 4, Table 5 and Table 6.

### 3.2. Compounds with Antioxidant and Healing Properties from Selected Caribean Plants

**Figure 2:**
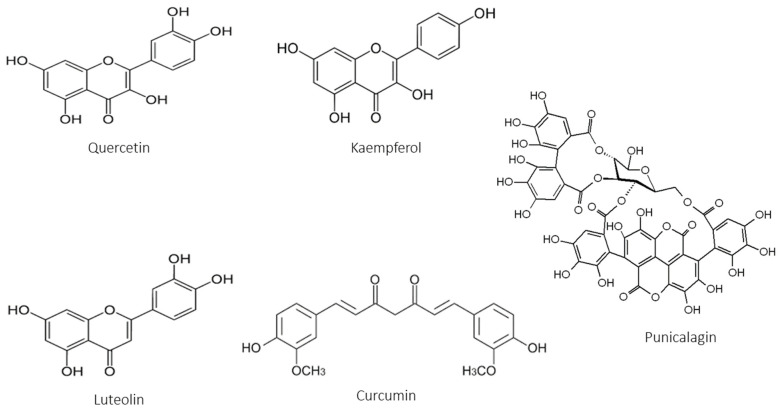
Polyphenols with beneficial properties for diabetic wound healing.

Many reviews have demonstrated the beneficial effect of different polyphenols on wound healing [157,158,159,160]. Interestingly, many molecules are found in the different extracts of Caribbean plants tested for their healing properties in diabetics. Quercetin or its derivatives, kaempferol and its derivatives, but also luteolin are molecules found in many extracts: we can therefore assume that these are the molecules responsible for the healing activity we obtain. Only curcumin and its derivatives have been identified in turmeric extract with healing properties. We will also evaluate the properties of punicalagin, a tannin that is re-found in pomegranate (Figure 2).

#### 3.2.1. Quercetin

Quercetin, or 3,3′,4′,5,7-Pentahydroxyflavone, is a flavonol found in a large number of fruits, vegetables, and plants. It is a polyphenol that has a large number of biological activities: antioxidant, anti-inflammatory, anti-cancer, and prevention of cardiovascular diseases [161]. Fu et al. evaluated the effect of quercetin on the wound healing of diabetic rats; it improved wound healing by improving wound contraction, fibroblast activity, and collagen deposition [162]. Interestingly, Fu et al. also looked at its effect on macrophages in the wound bed; quercetin increased the number of CD206-positive cells, a marker of M2 polarization, and decreased iNOS cells, a marker of M1 polarization. These results suggest that quercetin can regulate the polarization of macrophages from M1 to M2, which is in agreement with the results found with the level of proinflammatory cytokines. Furthermore, it has been described in different articles that quercetin can modulate the different targets involved in the defect of diabetic foot healing. Ganesan et al. demonstrated that quercetin inhibited the expression and activity of MMP9 and MMP12 in a mouse model of chronic obstructive pulmonary disease [163]. Bhuiyan et al. evaluated its effect on the formation of AGEs; it inhibits the accumulation of AGEs via the chelation of metal ions and the trapping of methylglyoxal and reactive oxygen species, which are reagents of the Maillard reaction [164]. In a model of diabetic retinopathy, quercetin inhibits the expression of NLRP3 inflammasome components in human retinal microvascular endothelial cells (HRMECs) under high glucose conditions [165]. In the mouse model of rheumatoid arthritis, quercetin inhibited NET formation by suppressing autophagy, suggesting that quercetin may be a therapeutic avenue in this disease [166]. Quercetin is known for its antioxidant properties: it is a potent scavenger of reactive oxygen species such as O_2_^•−^, NO^•^, and ONOO^−^ [161] and decreases the production of intracellular ROS [167]. In addition, it can increase the level of Nrf2 and thus induce the expression of antioxidant genes in HaCaT cells [168]. This antioxidant activity of quercetin could be responsible for all the effects observed on the targets involved in wound healing by decreasing intracellular ROS; quercetin could inhibit the signaling pathways involved in the proinflammatory state of wounds, notably NF-κB.

Moreover, quercetin is also present in the form of quercetin glycosides, i.e., derivatives of quercetin conjugated to sugars. Interestingly, Kim et al. and Zheng et al. demonstrated that quercetin glycosides have antioxidant activity [169,170].

#### 3.2.2. Kaempferol

Kaempferol or 5,7-trihydroxy-2(4-hydroxyphenyl)-4H-1-benzopyran-4-one is a flavonol found in many plants, fruits, or vegetables. It has many pharmacological properties, such as antioxidant, anti-inflammatory, anticancer, cardioprotective, and antidiabetic properties [171]. Özay et al. evaluated the healing properties of kaempferol in diabetic rats: it improves wound contraction and re-epithelialization, and increases hydroproline and collagen content [172]. Furthermore, in Huh7 and SK-Hep-1 liver cancer cells, kaempferol inhibits MMP9 activity and decreases the level of metalloproteinase 9 in Huh-7. Ronsisvalle et al. evaluated its ability to inhibit the formation of glycation end products: it has a small inhibitory capacity in the in vitro BSA/Fructose model [173]. It is commonly described that kaempferol is anti-inflammatory. Therefore, the use of this flavonol has been studied as a therapy to treat atherosclerosis. They were able to evaluate the use of kaempferol in a targeted manner on atherosclerotic plaques: kaempferol decreased inflammation due to macrophages by decreasing the secretion of pro-inflammatory cytokines and by inducing the repolarization of macrophages via the blocking of NF-κB signaling associated with ROS [174]. Furthermore, in glaucoma pathology, kaempferol decreased NLRP3 and NLRP1 inflammasome activation, which attenuates retinal ganglion cell death via inhibition of NF-κB and JNK pathways [175]. Kaempferol is known to decrease primary tumors and lung metastases in the mouse model of breast cancer. Zeng et al. demonstrated that kaempferol inhibited NET formation by decreasing the production of ROS derived from NADPH oxidase [176].

The antioxidant properties of kaempferol and some of the glycosides of kaempferol are known. Indeed, it is known to be a powerful scavenger of superoxides, hydroxyl radicals, and peroxynitrite ions. Moreover, it has the ability to inhibit the enzymes that generate ROS, such as Xanthine oxidase. It is also known for its ability to decrease the accumulation of intracellular ROS [171]. Interestingly, kaempferol increased the Nrf2 signaling pathway and increased catalase and glutathione activity, and decreased malondialdehyde activity in HUVEC cells [177].

The antioxidant activity of kaempferol could therefore be responsible for its effects on the different mechanisms involved in the delayed wound healing of diabetic patients.

#### 3.2.3. Luteolin

Luteolin, or 3,4,5,7-tetrahydroxy flavone, is a flavone that is present in many plants, fruits, and vegetables. It has many biological effects, such as anti-inflammatory, anti-allergic, and anti-cancer effects, but also pro-oxidant or antioxidant properties [178]. Chen et al. have demonstrated the ability of luteolin to improve wound healing in diabetic rats by accelerating re-epithelialization and collagen deposition [179]. Furthermore, luteolin inhibits MMP9 in vitro [180] and decreases the expression of MMP9 and MMP2 in a mouse model of colon carcinoma [181]. Moreover, luteolin decreases the formation of fluorescent AGEs in the HSA (human serum albumin)/glyoxal system [182]. Its anti-inflammatory activity can be explained by its capacity to modulate macrophage polarization. Indeed, Wang et al. put LPS-activated RAW264.7 cells in the presence of luteolin; it decreased the M1 polarization surface markers and increased the M2 surface markers, which is in agreement with their cytokine secretions [183]. Luteolin can also inhibit NLRP3 inflammasome activation and IL-1β secretion in J774A.1 macrophages by modulating ASC oligomerization [184]. Jablonska et al. were able to demonstrate that increased NETosis is involved in oral cavity squamous cell carcinoma, and luteolin reduces the ability of patients’ neutrophils to form NETs [185]. The antioxidant properties of luteolin are known: it is a H_2_O_2_ scavenger that decreases intracellular ROS. Moreover, it can inhibit pro-oxidant enzymes and increase the expression of antioxidant enzymes [186,187,188].

H_2_O_2_ scavenging by luteolin or the decrease in intracellular ROS could decrease the activation of the NLRP3 inflammasome and decrease the M1 polarization of macrophages in order to improve wound healing.

#### 3.2.4. Curcumin

Curcumin, or 1,7-bis(4-hydroxy-3-methoxyphenyl)-1,6-heptadiene-3,5-dione, and its derivatives demethoxycurcumin and bisdemethoxy-curcumin, are polyphenols called curcuminoids isolated from *Curcuma longa* rhizome [189]. It is known for its numerous anti-inflammatory, anti-cancer, and antioxidant properties [190]. Kant et al. studied the effect of curcumin on wound healing in diabetic rats: curcumin improved re-epithelialization with an improvement in granulation tissue, an increase in fibroblast proliferation, and collagen deposition. In addition, curcumin improved neoangiogenesis [191]. The review by Kumar et al., 2012, relates the regulatory activity of expression, secretion, and activity of various MMPs in various diseases [192]. In stomach ulcers, curcumin decreased the activity of MMP9, and in patients with angina pectoris, curcumin supplementation reduced the expression of MMP2 and MMP9 [192]. In addition, the inhibitory activity of curcumin on glycation end products was tested: curcumin and its derivatives decreased the formation of AGES in a BSA+ Fructose system. It also inhibited the formation of methylglyoxal-induced AGEs by scavenging it and decreased the formation of carboxymethullysine or CML, an AGE in HUVEC (human umbilical vein endothelial cells) [193,194,195,196]. Furthermore, curcumin diminuted the percentage of M1 macrophages and induced a high percentage of M2 macrophages with a significant release of IL-10 in RAW264.7 cells activated by titanium particles [197]. However, the review by Momtazi-Borojeni et al. presents curcumin as a modulator of macrophage polarization because it is also able to induce the expression of M1 cytokines [198]. Curcumin can also suppress the activation of the NLRP3 inflammasome in LPS-stimulated J774A.1 mouse macrophages: it decreased the secretion of IL-1β and decreased the activation of caspase 1 [199]. Zhu et al. were able to demonstrate that curcumin could attenuate hepatic ischemia-reperfusion injury by inhibiting NET formation through suppression of the MEK/ERK pathway [200]. Curcumin has been shown to inhibit NETosis induced by polybrominated diphenyl ethers and brominated organic pollutants by decreasing ROS. This decrease in ROS is associated with an improvement in Nrf2 translocation in the nucleus [201].

In addition, the antioxidant activity of curcumin is well known. It is a powerful scavenger of ROS, such as superoxide anions, hydroxyl radicals, and peroxinitrite ions [190]. It can also increase the activity of catalase, superoxide dismutase, and glutathione peroxidase in rats [202]. This increase can be explained by its ability to activate the Nrf2 signaling pathway [156].

The decrease in ROS induced by curcumin could therefore improve healing by inducing M2 polarization in macrophages or by decreasing the activity of MMPs.

#### 3.2.5. Punicalagin

Punicalagin, or 2,3-hexahydroxydiphenoyl-4,6-gallagyl-D-glucose, is a hydrolyzable tannin found in pomegranate (juice, skin, fruit, bark, and leaves) but also in *Terminalia catappa* L., *Terminalia chebula* Retz [203]. This molecule is known for its numerous pharmacological properties: anticancer, hepato-protective, antimicrobial or antiviral, and anti-inflammatory [204].

The ability of punicalagin to improve diabetic wound healing has not yet been evaluated. Nevertheless, punicalagin is known for its healing properties. Indeed, Kumar et al. were able to demonstrate the beneficial effect of punicalagin in a rat wound healing model. It improves wound contraction and decreases the time to re-epithelialization [205]. Furthermore, in the Hela cell line, a model of cervical cancer, punicalagin decreases the activity of the metalloproteinases MMP2 and MMP9 and increases the expression of their inhibitors TIMP2 [206]. It is also known to decrease the formation of glycation end products by trapping methylglyoxal, for example [207]. In a mouse model of collagen-induced arthritis, punicalagin inhibited joint inflammation and bone destruction by shifting M1 macrophages to the M2 phenotype. Indeed, the secretion of iNOS and pro-inflammatory cytokines was decreased, while the expression of M2 markers such as Arginase 1 and IL-10 was increased [208]. Furthermore, it was also demonstrated in this model that punicalagin decreases the expression of NLRP3 and caspase 1, which is concomitant to the decrease in the release of IL-1β and interleukin 18 [208]. Thus, it can be assumed that NLRP3 inflammasome activation was inhibited. Jung Lo et al. were able to demonstrate that pretreatment with punicalagin of LPS-treated murine BV2 microglia cells attenuated inflammation with a reduction in the secretion of the pro-inflammatory cytokines iNOS, IL-1β, and IL-6. There was also a decrease in NF-κB activity and inhibition of NLRP3 activation [209]. The antioxidant properties of punicalagin are known. Indeed, it is known to scavenge 2,2-diphenyl-1- picrylhydrazyl radical (DPPH) and hydrogen peroxide H_2_O_2_ [210,211]. It is also known to decrease the production of intracellular and mitochondrial ROS [209]. Furthermore, punicalagin enhances Nrf2 and HO-1 expression in RAW264.7 macrophages, where oxidative stress was induced by LPS. There is also a decrease in ROS formation and nitric oxide (NO) with an increase in SOD expression [212]. All these elements suggest that punicalagin would be a good candidate to improve wound healing in diabetic patients.

**Figure 3:**
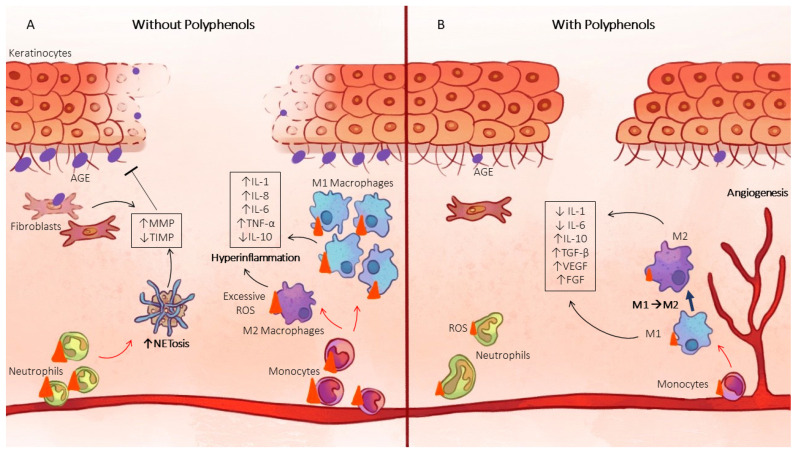
Polyphenols enhance diabetic wound healing. (**A**) Impaired wound healing in diabetic patients. Excessive ROS in diabetic patients induce an increase in inflammatory cell infiltration (neutrophils and monocytes/macrophages). Oxidative stress increase induces apoptosis of fibroblasts and keratinocytes. The pathology of diabetes induces an increase in the secretion of MMPs, which induces degradation of the extracellular matrix. Oxidative stress induces proinflammatory M1 hyperpolarization of macrophages and excessive secretion of proinflammatory mediators. Oxidative stress also promotes NETosis and the formation of glycation endproducts which also induce inflammation. The AGEs formed induce glycation of collagen in the extracellular matrix and inhibit the migration of keratinocytes. This results in a stop in the wound-healing process. (**B**) Improvement in diabetic wound healing with polyphenols. Polyphenols use reduces ROS quantity. This results in a decrease in the infiltration of pro-inflammatory cells (neutrophils and monocytes/macrophages) and inhibition of keratinocyte and fibroblast apoptosis. MMP secretion and activity are reduced. Polyphenols can also modulate macrophage polarization and promote anti-inflammatory M2 phenotype and inhibit NETosis.

Polyphenols, and in particular flavonoids and tannins, are known for their antioxidant properties. Quercetin, kaempferol, luteolin, and curcumin are known to trap ROS such as superoxide, H_2_O_2,_ and hydroxyl radicals but also peroxynitrite ions. They also decrease intracellular ROS. We can therefore assume that they can decrease the activation or even inhibit the signaling pathways activated by the increase in ROS in the healing of diabetic foot wounds. In addition, these molecules can also activate the transcription factor Nrf2, which will then trigger the transcription of antioxidant enzymes. Oxidative stress will be reduced; thus, the activity of overactivated MMPs will decrease, M1 macrophages will transition to an M2 phenotype, and the NLRP3 inflammasome will be inhibited, allowing the resolution of inflammation and the resumption of the healing process (Figure 3).

## 4. Future Directions

It is known that targeting ROS can improve wound healing. Indeed, Zhao et al. developed a hydrogel capable of trapping ROS, notably H_2_O_2_ and the hydroxyl radical. It can also decrease the level of intracellular ROS. This gel improved diabetic wound healing by decreasing oxidative stress and increasing the proportion of M2 macrophages [213]. The use of plants to improve wound healing seems to be a good strategy. Indeed, they are composed of molecules capable of either acting on the different mechanisms altered in patients or modulating oxidative stress in a fine way in order to promote healing.

Despite numerous results on animal models, very few clinical trials have been carried out [214,215,216]. It would be interesting to evaluate the different plant extracts on diabetic patients. Numerous studies have been able to evaluate the effect of flavonoids on wound healing, which have been summarized in various reviews [160,217]. Many studies have also been able to highlight their beneficial action on diabetic wound healing [172,179,218,219,220]. This pro-healing activity can be explained by the different actions that flavonoids can have. As described previously, flavonoids can modulate the activity of MMPs [163,181,221] and the activation of the NLRP3 inflammasome [152,165,184]. They are also known to modulate macrophage polarization [162,174,183] and can inhibit NETosis [176,185]. In addition, their antioxidant properties are well known [221,222], and they can also activate the transcription factor Nrf2 [168,177]. The healing activity of tannins is much less documented. A few studies have been able to demonstrate the beneficial effect of tannins on wound healing. Punicalagin, ellagic acid, or gallic acid are known to improve wound healing [205,223,224]. These tannins can both modulate the activity of MMPs [225,226] and NLRP3 activation, but also modulate macrophage polarization [227]. Moreover, tannins are also known for their antioxidant properties [228] and their ability to modulate the activity of the transcription factor Nrf2 [229,230].

Tannins, therefore, seem to be good candidates to improve wound healing in diabetic patients, and further research is needed to confirm this hypothesis. The antioxidant and healing activities of polyphenols are commonly studied; nevertheless, other types of secondary plant metabolites could carry out activities. Indeed, charantine, for example, is a triterpene that can be isolated from *Momordica charantia*. This molecule is known for its antioxidant and inhibitory properties on the formation of glycation end products [136]. In addition, triterpenes have many interesting properties for the healing of diabetic wounds. Indeed, lupeol, which is a triterpene able to decrease the activity of MMPs, decreases the activation of NLRP3 and modulates the polarization of macrophages [231,232,233]. In addition, lupeol activates Nrf2 signaling [234]. Therefore, it seems that triterpenes are also able to improve diabetic foot wound healing. This hypothesis is confirmed by Beserra et al., who evaluated the effect of lupeol on the healing of diabetic rats. In this study, they administered lupeol topically to diabetic rat wounds and observed an increase in the percentage of wound closure and contraction in the lupeol-treated group. There was also a decrease in the number of inflammatory cells and collagen deposition, with a decrease in IL-6 secretion and an increase in IL-10. It would therefore be interesting to evaluate the effect of different terpenes on wound healing [235].

## 5. Conclusions

The antioxidant strategy seems promising for improving the healing of diabetic foot wounds. Indeed, the increase in oxidative stress in diabetic patients could be responsible for the alteration of different mechanisms involved in wound healing, such as the activity of metalloproteinases, the activity of NLRP3, the polarization of macrophages, and the NETosis phenomenon. ROS also seems to play a role in the Nrf2/HO-1 pathway and the increase in glycation end products. The use of molecules or extracts from plants, natural sources of compounds with antioxidant properties, is, therefore, an interesting therapeutic avenue.

## Figures and Tables

**Table 1:** Selected plants improve the healing of diabetic wounds and the composition of extracts. STZ rats: streptozotocin diabetics rats; STZ/NAD rats: streptozotocin and nicotinamide diabetics rats; alloxan rats: alloxan diabetics rats; and alloxan mice: alloxan diabetics mice.

Scientific Name	Mechanism of Action	Extract	Model	Ref.	Extract Composition		Ref.
*Aloe vera*	- Increase collagen deposition - Decrease in re-epithelialization period	Gel extract	STZ rats	[96]	Apigenin	Quercetin	[97]
					Myricetin	Rutin	
					Isovitexin	Epicatechin	
					Kaempferol	p-coumarin	
					Luteolin	Cafeic acid	
					Ferulic acid		
*Annona Squamosa*	- Increase collagen deposition - Increase wound contraction - Decrease in re-epithelialization period	Ethanolic leaf extract	STZ rats	[98]	Rutin	Quercetin	[99,100]
					Kaempferol	Isorhamnetin	
*Carica papaya*	- Increase wound contraction-Increase wound contraction - Decrease in re-epithelialization period	Juice of unripe fruit	STZ rats	[94]	Caffeic acid	Ferulic acid	[101,102,103]
					p-coumaric acid	Quercetin	
					Rutin	Kaempferol	
					Myricetin		
*Curcuma longa*	- Increase in number of cells - Increase in number of cells - Decrease in re-epithelialization period	Aqueous extract	STZ rats	[104]	Curcumin		[105]
					Demethoxycurcumin		
					Bisdemethoxycurcumin		
*Momordica charantia*	- Improve re-epithelialization - Improve protein content - Increase collagen deposition	Aqueous extract of dried fruits	STZ rats	[106]	Caffeic acid	Catechin	[107,108]
					Epicatechin	Charantin	
	- Improve re-epithelialization - Improve protein content - Improve TGF-β1 expression	Aqueous fruit extract	STZ rats	[109]	Chlorogenic acid	Gallic acid	
					Ferulic acid		
*Moringa oleifera*	- Increase collagen deposition - Increase wound contraction - Increase neo-angiogenesis - Increase SOD and catalase activities - Decrease in re-epithelialization period	Methanolic extract of dried leaves	STZ rats	[110]	Quercetin glycoside Kaempferol glycoside Vicenin-2		[111]
	- Increase collagen deposition - Increase wound contraction - Increase neo-angiogenesis - Decrease in re-epithelialization period	Aqueous fraction of methanolic leaf extract	STZ and NAD rats	[112]			
	- Increase collagen - deposition - Increase wound contraction - Increase neo-angiogenesis	Aqueous extract of leaves	STZ rats		Citric acid	Quinic acid	[113]
				[114]	Vicenin-2	Vitexin	
					Quercetin glycoside	Luteolin glycoside	
*Psidium guajava* L.	- Increase wound contraction	Acetone extract of leaves	Alloxan rats	[115]	Guavanoic acid	guavacoumaric acid	[116]
					Myricetin	Quercetin	
					Luteolin	Kaempferol	
*Punica granatum*	- Increase collagen deposition - Increase neo-angiogenesis - Improve TGF-β1, VEGF, and EGF expression - Decrease in re-epithelialization period	Ethanolic extract of dried skin fruit	Alloxan rats	[117]	Punicalagin	Gallic acid	[118]
					Ellagic acid	Rutin	
					Quercetin		
*Rosmarinus officinalis*	- Improve granulation tissue - Improve wound contraction - Improve angiogenesis	Aqueous extract of the aerial part	Alloxan mice	[119]	Kaempferol glycoside	Quercetin glycoside	[120]
					Gallic acid	Quercetin	
					Gentisic acid	Epicatechin	
					Rutin	Ellagic acid	
					Rosmarinic acid		

**Table 2:** Effect of extracts of selected plants on metalloproteinases.

Plants	Extracts	Model	Effect	References
*Aloe vera*	Gel	LPS activated PBMC	Decrease MMP9 activity and expression	[121]
*Annona squamosa*	Leaves extract	Rats exposed to UVB	Decrease MMP1 expression	[126]
*Carica papaya*	Ethanolic dried leaves extract	NHDFs cells exposed to UVB	Decrease MMP1 and MMP3 expression	[127]
*Curcuma longa*	Curcuminoids	Keratinocytes HaCaT and Fibroblasts	Decrease MMP1 expression	[129]
*Momordica charantia*	Ethanolic leaves extract	PLS10 cells	Decrease MMP2 and MMP9 secretion	[135]
*Moringa oleifa*	Isothiocyanate of seeds	Keratinocytes HaCaT	Decrease MMP1, MMP3, and MMP9 expression	[140]
*Psidium guajava*	Aqueous leaves extract	DU-145 cells	Decrease MMP2 and MMP9 expression	[144]
*Punica granatum*	Aqueous peels extract	LPS-induced lung inflammation mouse model	Inhibition MMP2 and MMP9 activities	[147]
*Rosmarinus officinalis*	Aqueous extract	Fibroblast and 3d reconstructed skin model	Decrease MMP1 expression and activation	[153]

**Table 3:** Effect of selected plant extracts on AGEs formation. Inhibition of AGE formation in vitro was assessed by measuring the modulation by plant extracts of the fluorescence of AGEs obtained by reacting bovine serum albumin (BSA) or lysozyme with sugar (glucose or fructose) or dicarbonyl (methylglyoxal).

Plants	Extracts	Model	Effect	References
*Aloe vera*	Methanolic extract	BSA + Glucose	Inhibit AGE and N∊-Carboxymethyl-lysine (CML) formation	[122]
*Carica papaya*	Fruit juice	Aldolase reductase and Sorbitol dehydrogenase activity assay	Inhibit enzymes of the polyol pathway, which is involved in the formation of AGEs	[128]
*Curcuma longa*	Curcumin	Diabetic rats	Decrease AGE-Collagen	[130]
*Momordica charantia*	Methanolic extract	Lysozyme + Methylglyoxal	Inhibit AGE and CML formation	[136]
*Moringa oleifa*	Methanolic and aqueous extracts	BSA+ Fructose	Inhibit CML formation and crosslinking breaker capacity	[141]
*Psidium guajava* L.	Aqueous extract	BSA + Glucose	Inhibit AGE formation	[145]
*Punica granatum*	Fruit juice	BSA + Glucose/Fructose	Inhibit glyceraldehyde-derived AGE formation	[148]
*Rosmarinus officinalis*	Aqueous extract	BSA+ Fructose	Inhibit AGE formation	[151]

**Table 4:** Effect of extracts of selected plants on macrophage polarization.

Plants	Extracts	Model	Effect	References
*Aloe vera*	Gel	Rats	Modulate macrophages polarization	[123]
*Curcuma longa*	Gel	Mice and RAW264.7 macrophages	Enhance M2	[132]
*Momordica charantia*	Fruit powder	Mice	Enhance M2 and decrease M1	[155]
*Punica granatum*	Juice	J774A.1 macrophages	Enhance M2: increase IL-10 and decrease IL-6	[149]

**Table 5:** Effect of extracts of selected plants on NLRP3 inflammasome activation.

Plants	Extracts	Model	Effect	References
*Aloe vera*	Gel	THP1 macrophages	Decrease IL-1β and NLRP3 compounds Decrease NF-κB and MAPK activation	[124]
*Curcuma longa*	Curcumin	Depressive rats model	Inhibit NLRP3 Decrease mature IL-1β production	[133]
*Momordica charantia*	Acetonic fraction of fruit extract	RAW264.7 Mice	Decrease NLRP3 expression	[137,138]
*Moringa oleifa*	Ethanolic extract	Methotrexate-Induced Oxidative Stress and Apoptosis on Mouse Spleen	Decrease NLRP3 expression Increase antioxydant enzymes	[142]
*Psidium guajava*	Flavonoids leaf extract	Chronic Pancreatitis Mouse Model	Decrease NLRP3 expression Decrease IL-1β and IL-18 expression	[146]
*Rosmarinus officinalis*	Carnosic acid	Human macrophages	Inhibit NLRP3 activation Decrease mitochondrial ROS production	[152]

**Table 6:** Effect of extracts of selected plants on Nrf2 activation.

Plants	Extracts	Model	Effect	References
*Aloe vera*	Polysaccharides	Acute colitis in mice	Enhance Nrf2 expression	[125]
*Curcuma longa*	Curcumin	Lymphoma ascites cells	Enhance antioxydant enzyme expression Decrease proinflammatory cytokines	[134,156]
*Momordica charantia*	Polysaccharides	Hypothalamus rats	Increase antioxydant enzyme expression Improve Nrf2 translocation	[139]
*Moringa oleifa*	Aqueous leaves extract	Hepatic steatosis mices	Increase Nrf2 expression	[143]
*Punica granatum*	Fruit wine	SH-SY5Ycells	Increase Nrf2 translocation Inhibit NF-κB	[150]
*Rosmarinus officinalis*	Carnosol	HCT116 cells	Increase Nrf2 activation	[154]

## References

[1] Fédération Internationale du Diabète (2021). Atlas du Diabète de la FID.

[2] Chang M., Nguyen T.T. (2021). Strategy for Treatment of Infected Diabetic Foot Ulcers. Acc. Chem. Res..

[3] Fui L.W., Lok M.P.W., Govindasamy V., Yong T.K., Lek T.K., Das A.K. (2019). Understanding the Multifaceted Mechanisms of Diabetic Wound Healing and Therapeutic Application of Stem Cells Conditioned Medium in the Healing Process. J. Tissue Eng. Regen. Med..

[4] Zubair M., Ahmad J. (2019). Role of Growth Factors and Cytokines in Diabetic Foot Ulcer Healing: A Detailed Review. Rev. Endocr. Metab. Disord..

[5] Wong S.L., Demers M., Martinod K., Gallant M., Wang Y., Goldfine A.B., Kahn C.R., Wagner D.D. (2015). Diabetes Primes Neutrophils to Undergo NETosis Which Severely Impairs Wound Healing. Nat. Med..

[6] Zhao R., Liang H., Clarke E., Jackson C., Xue M., Zhao R., Liang H., Clarke E., Jackson C., Xue M. (2016). Inflammation in Chronic Wounds. Int. J. Mol. Sci..

[7] Bannon P., Wood S., Restivo T., Campbell L., Hardman M.J., Mace K.A. (2013). Diabetes Induces Stable Intrinsic Changes to Myeloid Cells That Contribute to Chronic Inflammation during Wound Healing in Mice. Dis. Model. Mech..

[8] Kasuya A., Tokura Y. (2014). Attempts to Accelerate Wound Healing. J. Dermatol. Sci..

[9] Ferrante C.J., Leibovich S.J. (2012). Regulation of Macrophage Polarization and Wound Healing. Adv. Wound Care.

[10] Boniakowski A.E., Kimball A.S., Jacobs B.N., Kunkel S.L., Gallagher K.A. (2017). Macrophage-Mediated Inflammation in Normal and Diabetic Wound Healing. J. Immunol..

[11] Dardenne C. (2015). Mécanismes de Dérégulation de la Polarisation des Macrophages et de la Résolution de L’inflammation au Cours de la Cicatrisation de Plaies Cutanées Chez des Souris Diabétiques de Type 2: Restauration par Application Topique D’aspirine. Ph.D. Thesis.

[12] Mirza R.E., Fang M.M., Weinheimer-Haus E.M., Ennis W.J., Koh T.J. (2014). Sustained Inflammasome Activity in Macrophages Impairs Wound Healing in Type 2 Diabetic Humans and Mice. Diabetes.

[13] Fadini G.P., Menegazzo L., Rigato M., Scattolini V., Poncina N., Bruttocao A., Ciciliot S., Mammano F., Ciubotaru C.D., Brocco E. (2016). NETosis Delays Diabetic Wound Healing in Mice and Humans. Diabetes.

[14] Wong S.L., Wagner D.D. (2018). Peptidylarginine Deiminase 4: A Nuclear Button Triggering Neutrophil Extracellular Traps in Inflammatory Diseases and Aging. FASEB J..

[15] Cano Sanchez M., Lancel S., Boulanger E., Neviere R. (2018). Targeting Oxidative Stress and Mitochondrial Dysfunction in the Treatment of Impaired Wound Healing: A Systematic Review. Antioxidants.

[16] Sen C.K., Roy S. (2008). Redox Signals in Wound Healing. Biochim. Biophys. Acta BBA—Gen. Subj..

[17] Schäfer M., Werner S. (2008). Oxidative Stress in Normal and Impaired Wound Repair. Pharmacol. Res..

[18] Bryan N., Ahswin H., Smart N., Bayon Y., Wohlert S., Hunt J. (2012). Reactive Oxygen Species (ROS)—A Family of Fate Deciding Molecules Pivotal in Constructive Inflammation and Wound Healing. Eur. Cell. Mater..

[19] Zhang P., Li T., Wu X., Nice E.C., Huang C., Zhang Y. (2020). Oxidative Stress and Diabetes: Antioxidative Strategies. Front. Med..

[20] Evans J.L., Goldfine I.D., Maddux B.A., Grodsky G.M. (2002). Oxidative Stress and Stress-Activated Signaling Pathways: A Unifying Hypothesis of Type 2 Diabetes. Endocr. Rev..

[21] Monnier L., Mas E., Ginet C., Michel F., Villon L., Cristol J.-P., Colette C. (2006). Activation of Oxidative Stress by Acute Glucose Fluctuations Compared With Sustained Chronic Hyperglycemia in Patients With Type 2 Diabetes. JAMA.

[22] Wei W., Liu Q., Tan Y., Liu L., Li X., Cai L. (2009). Oxidative Stress, Diabetes, and Diabetic Complications. Hemoglobin.

[23] Wlaschek M., Scharffetter-Kochanek K. (2005). Oxidative Stress in Chronic Venous Leg Ulcers. Wound Repair Regen..

[24] Deng L., Du C., Song P., Chen T., Rui S., Armstrong D.G., Deng W. (2021). The Role of Oxidative Stress and Antioxidants in Diabetic Wound Healing. Oxid. Med. Cell. Longev..

[25] Song J., Liu A., Liu B., Huang W., Jiang Z., Bai X., Hu L., Zheng S., Guo S., Wu J. (2022). Natural Biologics Accelerate Healing of Diabetic Foot Ulcers by Regulating Oxidative Stress. Front. Biosci.-Landmark.

[26] Dhall S., Do D.C., Garcia M., Kim J., Mirebrahim S.H., Lyubovitsky J., Lonardi S., Nothnagel E.A., Schiller N., Martins-Green M. (2014). Generating and Reversing Chronic Wounds in Diabetic Mice by Manipulating Wound Redox Parameters. J. Diabetes Res..

[27] Park J.Y., Shin M.-S., Hwang G.S., Yamabe N., Yoo J.-E., Kang K.S., Kim J.-C., Lee J.G., Ham J., Lee H.L. (2018). Beneficial Effects of Deoxyshikonin on Delayed Wound Healing in Diabetic Mice. Int. J. Mol. Sci..

[28] Yan J., Tie G., Wang S., Tutto A., DeMarco N., Khair L., Fazzio T.G., Messina L.M. (2018). Diabetes Impairs Wound Healing by Dnmt1-Dependent Dysregulation of Hematopoietic Stem Cells Differentiation towards Macrophages. Nat. Commun..

[29] Khanna S., Biswas S., Shang Y., Collard E., Azad A., Kauh C., Bhasker V., Gordillo G.M., Sen C.K., Roy S. (2010). Macrophage Dysfunction Impairs Resolution of Inflammation in the Wounds of Diabetic Mice. PLoS ONE.

[30] Lan C.-C.E., Wu C.-S., Huang S.-M., Wu I.-H., Chen G.-S. (2013). High-Glucose Environment Enhanced Oxidative Stress and Increased Interleukin-8 Secretion from Keratinocytes: New Insights into Impaired Diabetic Wound Healing. Diabetes.

[31] Pustovrh M.C., Jawerbaum A., Capobianco E., White V., Martínez N., López-Costa J.J., González E. (2005). Oxidative Stress Promotes the Increase of Matrix Metalloproteinases-2 and -9 Activities in the Feto-Placental Unit of Diabetic Rats. Free Radic. Res..

[32] Rajagopalan S., Meng X.P., Ramasamy S., Harrison D.G., Galis Z.S. (1996). Reactive Oxygen Species Produced by Macrophage-Derived Foam Cells Regulate the Activity of Vascular Matrix Metalloproteinases in Vitro. Implications for Atherosclerotic Plaque Stability. J. Clin. Investig..

[33] Loo A.E.K., Wong Y.T., Ho R., Wasser M., Du T., Ng W.T., Halliwell B. (2012). Effects of Hydrogen Peroxide on Wound Healing in Mice in Relation to Oxidative Damage. PLoS ONE.

[34] Kunkemoeller B., Kyriakides T.R. (2017). Redox Signaling in Diabetic Wound Healing Regulates Extracellular Matrix Deposition. Antioxid. Redox Signal..

[35] Zhang W., Chen L., Xiong Y., Panayi A.C., Abududilibaier A., Hu Y., Yu C., Zhou W., Sun Y., Liu M. (2021). Antioxidant Therapy and Antioxidant-Related Bionanomaterials in Diabetic Wound Healing. Front. Bioeng. Biotechnol..

[36] Jones J.I., Nguyen T.T., Peng Z., Chang M. (2019). Targeting MMP-9 in Diabetic Foot Ulcers. Pharmaceuticals.

[37] Choma P. (2019). Développement d’un Pansement Intelligent par Détection de Metalloprotéases. Ph.D. Thesis.

[38] Liang Y., Yang C., Lin Y., Parviz Y., Sun K., Wang W., Ren M., Yan L. (2019). Matrix Metalloproteinase 9 Induces Keratinocyte Apoptosis through FasL/Fas Pathway in Diabetic Wound. Apoptosis.

[39] Nelson K.K., Melendez J.A. (2004). Mitochondrial Redox Control of Matrix Metalloproteinases. Free Radic. Biol. Med..

[40] Belkhiri A., Richards C., Whaley M., McQueen S.A., Orr F.W. (1997). Increased Expression of Activated Matrix Metalloproteinase-2 by Human Endothelial Cells after Sublethal H2O2 Exposure. Lab. Investig. J. Tech. Methods Pathol..

[41] Hsieh H.-L., Lin C.-C., Hsiao L.-D., Yang C.-M. (2013). High Glucose Induces Reactive Oxygen Species-Dependent Matrix Metalloproteinase-9 Expression and Cell Migration in Brain Astrocytes. Mol. Neurobiol..

[42] Kowluru R.A., Shan Y. (2017). Role of Oxidative Stress in Epigenetic Modification of MMP-9 Promoter in the Development of Diabetic Retinopathy. Graefes Arch. Clin. Exp. Ophthalmol..

[43] Nam H., Kim M.-M. (2013). Eugenol with Antioxidant Activity Inhibits MMP-9 Related to Metastasis in Human Fibrosarcoma Cells. Food Chem. Toxicol..

[44] Nguyen T.T., Ding D., Wolter W.R., Pérez R.L., Champion M.M., Mahasenan K.V., Hesek D., Lee M., Schroeder V.A., Jones J.I. (2018). Validation of Matrix Metalloproteinase-9 (MMP-9) as a Novel Target for Treatment of Diabetic Foot Ulcers in Humans and Discovery of a Potent and Selective Small-Molecule MMP-9 Inhibitor That Accelerates Healing. J. Med. Chem..

[45] Vistoli G., De Maddis D., Cipak A., Zarkovic N., Carini M., Aldini G. (2013). Advanced Glycoxidation and Lipoxidation End Products (AGEs and ALEs): An Overview of Their Mechanisms of Formation. Free Radic. Res..

[46] Morita K., Urabe K., Moroi Y., Koga T., Nagai R., Horiuchi S., Furue M. (2005). Migration of Keratinocytes Is Impaired on Glycated Collagen I. Wound Repair Regen..

[47] Zhu P., Yang C., Chen L.-H., Ren M., Lao G., Yan L. (2011). Impairment of Human Keratinocyte Mobility and Proliferation by Advanced Glycation End Products-Modified BSA. Arch. Dermatol. Res..

[48] Guimarães E.L.M., Empsen C., Geerts A., van Grunsven L.A. (2010). Advanced Glycation End Products Induce Production of Reactive Oxygen Species via the Activation of NADPH Oxidase in Murine Hepatic Stellate Cells. J. Hepatol..

[49] Nowotny K., Jung T., Höhn A., Weber D., Grune T. (2015). Advanced Glycation End Products and Oxidative Stress in Type 2 Diabetes Mellitus. Biomolecules.

[50] Schmidt A.M., Yan S.D., Yan S.F., Stern D.M. (2001). The Multiligand Receptor RAGE as a Progression Factor Amplifying Immune and Inflammatory Responses. J. Clin. Investig..

[51] Ott C., Jacobs K., Haucke E., Navarrete Santos A., Grune T., Simm A. (2014). Role of Advanced Glycation End Products in Cellular Signaling. Redox Biol..

[52] Peppa M., Stavroulakis P., Raptis S.A. (2009). Advanced Glycoxidation Products and Impaired Diabetic Wound Healing. Wound Repair Regen..

[53] Shaikh-Kader A., Houreld N.N., Rajendran N.K., Abrahamse H. (2019). The Link between Advanced Glycation End Products and Apoptosis in Delayed Wound Healing. Cell Biochem. Funct..

[54] Miyata T., Wada Y., Cai Z., Iida Y., Horie K., Yasuda Y., Maeda K., Kurokawa K., De Strihou C.V.Y. (1997). Implication of an Increased Oxidative Stress in the Formation of Advanced Glycation End Products in Patients with End-Stage Renal Failure. Kidney Int..

[55] Xu F., Zhang C., Graves D.T. (2013). Abnormal Cell Responses and Role of TNF-α in Impaired Diabetic Wound Healing. BioMed Res. Int..

[56] Rendra E., Riabov V., Mossel D.M., Sevastyanova T., Harmsen M.C., Kzhyshkowska J. (2019). Reactive Oxygen Species (ROS) in Macrophage Activation and Function in Diabetes. Immunobiology.

[57] Aitcheson S.M., Frentiu F.D., Hurn S.E., Edwards K., Murray R.Z. (2021). Skin Wound Healing: Normal Macrophage Function and Macrophage Dysfunction in Diabetic Wounds. Molecules.

[58] Zhang Y., Choksi S., Chen K., Pobezinskaya Y., Linnoila I., Liu Z.-G. (2013). ROS Play a Critical Role in the Differentiation of Alternatively Activated Macrophages and the Occurrence of Tumor-Associated Macrophages. Cell Res..

[59] Mirza R., Koh T.J. (2011). Dysregulation of Monocyte/Macrophage Phenotype in Wounds of Diabetic Mice. Cytokine.

[60] Zhang B., Yang Y., Yi J., Zhao Z., Ye R. (2021). Hyperglycemia Modulates M1/M2 Macrophage Polarization via Reactive Oxygen Species Overproduction in Ligature-Induced Periodontitis. J. Periodontal Res..

[61] Pérez S., Rius-Pérez S. (2022). Macrophage Polarization and Reprogramming in Acute Inflammation: A Redox Perspective. Antioxidants.

[62] Covarrubias A., Byles V., Horng T. (2013). ROS Sets the Stage for Macrophage Differentiation. Cell Res..

[63] He Y., Hara H., Núñez G. (2016). Mechanism and Regulation of NLRP3 Inflammasome Activation. Trends Biochem. Sci..

[64] Jo E.-K., Kim J.K., Shin D.-M., Sasakawa C. (2016). Molecular Mechanisms Regulating NLRP3 Inflammasome Activation. Cell. Mol. Immunol..

[65] Abais J.M., Xia M., Zhang Y., Boini K.M., Li P.-L. (2015). Redox Regulation of NLRP3 Inflammasomes: ROS as Trigger or Effector?. Antioxid. Redox Signal..

[66] Dai J., Chen H., Chai Y. (2019). Advanced Glycation End Products (AGEs) Induce Apoptosis of Fibroblasts by Activation of NLRP3 Inflammasome via Reactive Oxygen Species (ROS) Signaling Pathway. Med. Sci. Monit. Int. Med. J. Exp. Clin. Res..

[67] Zhang X., Dai J., Li L., Chen H., Chai Y. (2017). NLRP3 Inflammasome Expression and Signaling in Human Diabetic Wounds and in High Glucose Induced Macrophages. J. Diabetes Res..

[68] Ding Y., Ding X., Zhang H., Li S., Yang P., Tan Q. (2022). Relevance of NLRP3 Inflammasome-Related Pathways in the Pathology of Diabetic Wound Healing and Possible Therapeutic Targets. Oxid. Med. Cell. Longev..

[69] Brinkmann V., Reichard U., Goosmann C., Fauler B., Uhlemann Y., Weiss D.S., Weinrauch Y., Zychlinsky A. (2004). Neutrophil Extracellular Traps Kill Bacteria. Science.

[70] Vorobjeva N.V., Chernyak B.V. (2020). NETosis: Molecular Mechanisms, Role in Physiology and Pathology. Biochem. Mosc..

[71] Karima M., Kantarci A., Ohira T., Hasturk H., Jones V.L., Nam B.-H., Malabanan A., Trackman P.C., Badwey J.A., Van Dyke T.E. (2005). Enhanced Superoxide Release and Elevated Protein Kinase C Activity in Neutrophils from Diabetic Patients: Association with Periodontitis. J. Leukoc. Biol..

[72] Dovi J.V., He L.-K., DiPietro L.A. (2003). Accelerated Wound Closure in Neutrophil-Depleted Mice. J. Leukoc. Biol..

[73] Wang L., Zhou X., Yin Y., Mai Y., Wang D., Zhang X. (2019). Hyperglycemia Induces Neutrophil Extracellular Traps Formation Through an NADPH Oxidase-Dependent Pathway in Diabetic Retinopathy. Front. Immunol..

[74] Yang C., Chen L., Chen W., Li N., Chen M., Li X., Zheng X., Zhao Y., Wu Y., Xian M. (2019). Hydrogen Sulfide Primes Diabetic Wound to Close through Inhibition of NETosis. Mol. Cell. Endocrinol..

[75] Lee M.K.S., Sreejit G., Nagareddy P.R., Murphy A.J. (2020). Attack of the NETs! NETosis Primes IL-1β-Mediated Inflammation in Diabetic Foot Ulcers. Clin. Sci..

[76] Liu D., Yang P., Gao M., Yu T., Shi Y., Zhang M., Yao M., Liu Y., Zhang X. (2019). NLRP3 Activation Induced by Neutrophil Extracellular Traps Sustains Inflammatory Response in the Diabetic Wound. Clin. Sci..

[77] Nakazawa D., Shida H., Kusunoki Y., Miyoshi A., Nishio S., Tomaru U., Atsumi T., Ishizu A. (2016). The Responses of Macrophages in Interaction with Neutrophils That Undergo NETosis. J. Autoimmun..

[78] Liu Y., Yang X., Liu Y., Jiang T., Ren S., Chen J., Xiong H., Yuan M., Li W., Machens H.-G. (2021). NRF2 Signalling Pathway: New Insights and Progress in the Field of Wound Healing. J. Cell. Mol. Med..

[79] Süntar I., Çetinkaya S., Panieri E., Saha S., Buttari B., Profumo E., Saso L. (2021). Regulatory Role of Nrf2 Signaling Pathway in Wound Healing Process. Molecules.

[80] Kansanen E., Kuosmanen S.M., Leinonen H., Levonen A.-L. (2013). The Keap1-Nrf2 Pathway: Mechanisms of Activation and Dysregulation in Cancer. Redox Biol..

[81] Loboda A., Damulewicz M., Pyza E., Jozkowicz A., Dulak J. (2016). Role of Nrf2/HO-1 System in Development, Oxidative Stress Response and Diseases: An Evolutionarily Conserved Mechanism. Cell. Mol. Life Sci..

[82] Long M., Rojo de la Vega M., Wen Q., Bharara M., Jiang T., Zhang R., Zhou S., Wong P.K., Wondrak G.T., Zheng H. (2016). An Essential Role of NRF2 in Diabetic Wound Healing. Diabetes.

[83] Jindam A., Yerra V.G., Kumar A. (2017). Nrf2: A Promising Trove for Diabetic Wound Healing. Ann. Transl. Med..

[84] Soares M.A., Cohen O.D., Low Y.C., Sartor R.A., Ellison T., Anil U., Anzai L., Chang J.B., Saadeh P.B., Rabbani P.S. (2016). Restoration of Nrf2 Signaling Normalizes the Regenerative Niche. Diabetes.

[85] Rabbani P.S., Zhou A., Borab Z.M., Frezzo J.A., Srivastava N., More H.T., Rifkin W.J., David J.A., Berens S.J., Chen R. (2017). Novel Lipoproteoplex Delivers Keap1 SiRNA Based Gene Therapy to Accelerate Diabetic Wound Healing. Biomaterials.

[86] Grochot-Przeczek A., Lach R., Mis J., Skrzypek K., Gozdecka M., Sroczynska P., Dubiel M., Rutkowski A., Kozakowska M., Zagorska A. (2009). Heme Oxygenase-1 Accelerates Cutaneous Wound Healing in Mice. PLoS ONE.

[87] Bitar M.S., Al-Mulla F. (2011). A Defect in Nrf2 Signaling Constitutes a Mechanism for Cellular Stress Hypersensitivity in a Genetic Rat Model of Type 2 Diabetes. Am. J. Physiol. Endocrinol. Metab..

[88] Shedoeva A., Leavesley D., Upton Z., Fan C. (2019). Wound Healing and the Use of Medicinal Plants. Evid.-Based Complement. Altern. Med. ECAM.

[89] Firdous S.M., Sautya D. (2018). Medicinal Plants with Wound Healing Potential. Bangladesh J. Pharmacol..

[90] Budovsky A., Yarmolinsky L., Ben-Shabat S. (2015). Effect of Medicinal Plants on Wound Healing. Wound Repair Regen. Off. Publ. Wound Heal. Soc. Eur. Tissue Repair Soc..

[91] Oguntibeju O.O. (2019). Medicinal Plants and Their Effects on Diabetic Wound Healing. Vet. World.

[92] Ande S.N., Dhandar K.M., Bakal R.L. (2022). Medicinal Herbs: Why to Include in Diabetic Foot Ulcer Therapy? A Review. Innov. Pharm. Pharmacother..

[93] Herman A., Herman A.P. (2023). Herbal Products and Their Active Constituents for Diabetic Wound Healing—Preclinical and Clinical Studies: A Systematic Review. Pharmaceutics.

[94] Nayak S.B., Pinto Pereira L., Maharaj D. (2007). Wound Healing Activity of Carica Papaya L. in Experimentally Induced Diabetic Rats. Indian J. Exp. Biol..

[95] Fournet J. (2002). Flore Illustrée des Phanérogames de Guadeloupe et de Martinique/Jacques Fournet.

[96] Chithra P., Sajithlal G.B., Chandrakasan G. (1998). Influence of Aloe Vera on the Healing of Dermal Wounds in Diabetic Rats. J. Ethnopharmacol..

[97] Kahramanoğlu İ., Chen C., Chen J., Wan C. (2019). Chemical Constituents, Antimicrobial Activity, and Food Preservative Characteristics of Aloe Vera Gel. Agronomy.

[98] Ponrasu T., Suguna L. (2012). Efficacy of *Annona squamosa* on Wound Healing in Streptozotocin-Induced Diabetic Rats. Int. Wound J..

[99] Varadharaj V., Kumba U., Krishnamurthy V. (2012). Physicochemical, Phytochemical Screening and Profiling of Secondary Metabolites of *Annona squamosa* Leaf Extract. World J. Pharm. Res..

[100] Kumar M., Changan S., Tomar M., Prajapati U., Saurabh V., Hasan M., Sasi M., Maheshwari C., Singh S., Dhumal S. (2021). Custard Apple (*Annona squamosa* L.) Leaves: Nutritional Composition, Phytochemical Profile, and Health-Promoting Biological Activities. Biomolecules.

[101] Sharma A., Bachheti A., Sharma P., Bachheti R.K., Husen A. (2020). Phytochemistry, Pharmacological Activities, Nanoparticle Fabrication, Commercial Products and Waste Utilization of Carica Papaya L.: A Comprehensive Review. Curr. Res. Biotechnol..

[102] Khadam S., Afzal U., Gul H., Hira S., Satti M., Yaqub A., Ajab H., Gulfraz M. (2019). Phytochemical Screening and Bioactivity Assessment of Leaves and Fruits Extract of Carica Papaya. Pak. J. Pharm. Sci..

[103] Gayosso-García Sancho L.E., Yahia E.M., González-Aguilar G.A. (2011). Identification and Quantification of Phenols, Carotenoids, and Vitamin C from Papaya (Carica Papaya L., Cv. Maradol) Fruit Determined by HPLC-DAD-MS/MS-ESI. Food Res. Int..

[104] Sidhu G.S., Mani H., Gaddipati J.P., Singh A.K., Seth P., Banaudha K.K., Patnaik G.K., Maheshwari R.K. (1999). Curcumin Enhances Wound Healing in Streptozotocin Induced Diabetic Rats and Genetically Diabetic Mice. Wound Repair Regen..

[105] Niranjan A., Prof D. (2008). Chemical Constituents and Biological Activities of Turmeric (*Curcuma longa* L.)—A Review. J. Food Sci. Technol..

[106] Teoh S.L., Latiff A.A., Das S. (2009). The Effect of Topical Extract of Momordica Charantia (Bitter Gourd) on Wound Healing in Nondiabetic Rats and in Rats with Diabetes Induced by Streptozotocin. Clin. Exp. Dermatol..

[107] La Torre V., Guarniz W., Silva-Correa C., Razco L., Siche R. (2020). Antimicrobial Activity and Chemical Composition of Momordica Charantia: A Review. Pharmacogn. J..

[108] Liu J.-Q., Chen J.-C., Wang C.-F., Qiu M.-H. (2009). New Cucurbitane Triterpenoids and Steroidal Glycoside from Momordica Charantia. Molecules.

[109] Hussan F., Teoh S.L., Muhamad N., Mazlan M., Latiff A.A. (2014). Momordica Charantia Ointment Accelerates Diabetic Wound Healing and Enhances Transforming Growth Factor-β Expression. J. Wound Care.

[110] Al-Ghanayem A.A., Alhussaini M.S., Asad M., Joseph B. (2022). Moringa Oleifera Leaf Extract Promotes Healing of Infected Wounds in Diabetic Rats: Evidence of Antimicrobial, Antioxidant and Proliferative Properties. Pharmaceuticals.

[111] Muhammad A.A., Pauzi N.A.S., Arulselvan P., Abas F., Fakurazi S. (2013). In Vitro Wound Healing Potential and Identification of Bioactive Compounds from *Moringa Oleifera* Lam. BioMed Res. Int..

[112] Muhammad A.A., Arulselvan P., Cheah P.S., Abas F., Fakurazi S. (2016). Evaluation of Wound Healing Properties of Bioactive Aqueous Fraction from Moringa Oleifera Lam on Experimentally Induced Diabetic Animal Model. Drug Des. Dev. Ther..

[113] Braham F., Carvalho D.O., Almeida C.M.R., Zaidi F., Magalhães J.M.C.S., Guido L.F., Gonçalves M.P. (2020). Online HPLC-DPPH Screening Method for Evaluation of Radical Scavenging Phenols Extracted from Moringa Oleifera Leaves. S. Afr. J. Bot..

[114] Azevedo Í.M., Araújo-Filho I., Teixeira M.M.A., Moreira M.D.F.D.C., Medeiros A.C. (2018). Wound Healing of Diabetic Rats Treated with *Moringa Oleifera* Extract. Acta Cirúrgica Bras..

[115] Kumari J., Sangeetha M., Ali S. (2018). Formulation and Evaluation of Herbal Gel from Tannin-Enriched Fraction of *Psidium guajava* Linn. Leaves for Diabetic Wound Healing. Int. J. Green Pharm. IJGP.

[116] Gutiérrez R.M.P., Mitchell S., Solis R.V. (2008). Psidium Guajava: A Review of Its Traditional Uses, Phytochemistry and Pharmacology. J. Ethnopharmacol..

[117] Yan H., Peng K., Wang Q., Gu Z., Lu Y., Zhao J., Xu F. (2013). Effect of Pomegranate Peel Polyphenol Gel on Cutaneous Wound Healing in Alloxan-Induced Diabetic Rats. Chin. Med. J..

[118] Middha S.K., Usha T., Pande V. (2013). HPLC Evaluation of Phenolic Profile, Nutritive Content, and Antioxidant Capacity of Extracts Obtained from *Punica granatum* Fruit Peel. Adv. Pharmacol. Pharm. Sci..

[119] Abu-Al-Basal M.A. (2010). Healing Potential of Rosmarinus Officinalis L. on Full-Thickness Excision Cutaneous Wounds in Alloxan-Induced-Diabetic BALB/c Mice. J. Ethnopharmacol..

[120] Jena J., Gupta A.K. (2012). Ricinus communis linn: A phytopharmacological review. Int. J. Pharm. Pharm. Sci..

[121] Vijayalakshmi D., Dhandapani R., Jayaveni S., Jithendra P.S., Rose C., Mandal A.B. (2012). In Vitro Anti Inflammatory Activity of Aloe Vera by down Regulation of MMP-9 in Peripheral Blood Mononuclear Cells. J. Ethnopharmacol..

[122] Kang S., Zhao X., Yue L., Liu L. (2017). Main Anthraquinone Components in Aloe Vera and Their Inhibitory Effects on the Formation of Advanced Glycation End-Products. J. Food Process. Preserv..

[123] Ashouri F., Beyranvand F., Beigi Boroujeni N., Tavafi M., Sheikhian A., Varzi A.M., Shahrokhi S. (2019). Macrophage Polarization in Wound Healing: Role of Aloe Vera/Chitosan Nanohydrogel. Drug Deliv. Transl. Res..

[124] Budai M.M., Varga A., Milesz S., Tőzsér J., Benkő S. (2013). Aloe Vera Downregulates LPS-Induced Inflammatory Cytokine Production and Expression of NLRP3 Inflammasome in Human Macrophages. Mol. Immunol..

[125] Liu C., Hua H., Zhu H., Cheng Y., Guo Y., Yao W., Qian H. (2021). Aloe Polysaccharides Ameliorate Acute Colitis in Mice via Nrf2/HO-1 Signaling Pathway and Short-Chain Fatty Acids Metabolism. Int. J. Biol. Macromol..

[126] Natalia F., Pinatih N.I., Praharsini G.A.A. (2023). Administration of Sugar Apple Leaf Extract Cream (*Annona squamosa* L.) Increased Superoxide Dismutase (SOD) Activity and Decreased Skin Matrix Metalloproteinase-1 (MMP-1) Activity in Male White Rats (Rattus Norvegicus) Wistar Strain Exposed to Ultraviolet B Light. Int. J. Res. Rev..

[127] Seo S.A., Ngo H.T.T., Hwang E., Park B., Yi T.-H. (2020). Protective Effects of Carica Papaya Leaf against Skin Photodamage by Blocking Production of Matrix Metalloproteinases and Collagen Degradation in UVB-Irradiated Normal Human Dermal Fibroblasts. S. Afr. J. Bot..

[128] Kazeem M.I., Adeyemi A.A., Adenowo A.F., Akinsanya M.A. (2020). Carica Papaya Linn. Fruit Extract Inhibited the Activities of Aldose Reductase and Sorbitol Dehydrogenase: Possible Mechanism for Amelioration of Diabetic Complications. Future J. Pharm. Sci..

[129] Jang S., Chun J., Shin E.M., Kim H., Kim Y.S. (2012). Inhibitory Effects of Curcuminoids from *Curcuma longa* on Matrix Metalloproteinase-1 Expression in Keratinocytes and Fibroblasts. J. Pharm. Investig..

[130] Sajithlal G.B., Chithra P., Chandrakasan G. (1998). Effect of Curcumin on the Advanced Glycation and Cross-Linking of Collagen in Diabetic Rats. Biochem. Pharmacol..

[131] Afiat B., Hendi A., Andromeda A., Alexander K., Almahitta Cintami P., Nur A. (2020). Topical Administration of *Curcuma longa* L. Extract Gel Increases M2 Macrophage Polarization and Collagen Density in Skin Excision. J. Appl. Pharm. Sci..

[132] Gao S., Zhou J., Liu N., Wang L., Gao Q., Wu Y., Zhao Q., Liu P., Wang S., Liu Y. (2015). Curcumin Induces M2 Macrophage Polarization by Secretion IL-4 and/or IL-13. J. Mol. Cell. Cardiol..

[133] Zhang W., Guo Y., Han W., Yang M., Wen L., Wang K., Jiang P. (2019). Curcumin Relieves Depressive-like Behaviors via Inhibition of the NLRP3 Inflammasome and Kynurenine Pathway in Rats Suffering from Chronic Unpredictable Mild Stress. Int. Immunopharmacol..

[134] Das L., Vinayak M. (2018). Curcumin Modulates Glycolytic Metabolism and Inflammatory Cytokines via Nrf 2 in Dalton’s Lymphoma Ascites Cells In Vivo. Anticancer Agents Med. Chem..

[135] Pitchakarn P., Ogawa K., Suzuki S., Takahashi S., Asamoto M., Chewonarin T., Limtrakul P., Shirai T. (2010). Momordica Charantia Leaf Extract Suppresses Rat Prostate Cancer Progression in Vitro and in Vivo. Cancer Sci..

[136] Aljohi A., Matou-Nasri S., Ahmed N. (2016). Antiglycation and Antioxidant Properties of Momordica charantia. PLoS ONE.

[137] Perez J.L., Shivanagoudra S.R., Perera W.H., Kim D.M., Wu C.S., Sun Y., Jayaprakasha G.K., Patil B.S. (2021). Bitter Melon Extracts and Cucurbitane-Type Triterpenoid Glycosides Antagonize Lipopolysaccharide-Induced Inflammation via Suppression of NLRP3 Inflammasome. J. Funct. Foods.

[138] Nerurkar P.V., Orias D., Soares N., Kumar M., Nerurkar V.R. (2019). Momordica Charantia (Bitter Melon) Modulates Adipose Tissue Inflammasome Gene Expression and Adipose-Gut Inflammatory Cross Talk in High-Fat Diet (HFD)-Fed Mice. J. Nutr. Biochem..

[139] Yue J., Guo P., Jin Y., Li M., Hu X., Wang W., Wei X., Qi S. (2022). Momordica Charantia Polysaccharide Ameliorates D-Galactose-Induced Aging through the Nrf2/β-Catenin Signaling Pathway. Metab. Brain Dis..

[140] Wang Y., Ouyang Q., Chang X., Yang M., He J., Tian Y., Sheng J. (2022). Anti-Photoaging Effects of Flexible Nanoliposomes Encapsulated Moringa Oleifera Lam. Isothiocyanate in UVB-Induced Cell Damage in HaCaT Cells. Drug Deliv..

[141] Adeniran O.I., Mogale M.A. (2021). Inhibitory Effect and Cross-Link Breaking Activity of Moringa Oleifera Leaf Crude Extracts on Fructose-Derived Advanced Glycation End-Products. S. Afr. J. Bot..

[142] Soliman M.M., Al-Osaimi S.H., HassanMohamed E., Aldhahrani A., Alkhedaide A., Althobaiti F., Mohamed W.A. (2020). Protective Impacts of Moringa Oleifera Leaf Extract against Methotrexate-Induced Oxidative Stress and Apoptosis on Mouse Spleen. Evid. Based Complement. Altern. Med..

[143] Kim C.G., Chang S.N., Park S.M., Hwang B.S., Kang S.-A., Kim K.S., Park J.G. (2022). Moringa Oleifera Mitigates Ethanol-Induced Oxidative Stress, Fatty Degeneration and Hepatic Steatosis by Promoting Nrf2 in Mice. Phytomedicine.

[144] Peng C.-C., Peng C.-H., Chen K.-C., Hsieh C.-L., Peng R.Y. (2011). The Aqueous Soluble Polyphenolic Fraction of *Psidium guajava* Leaves Exhibits Potent Anti-Angiogenesis and Anti-Migration Actions on DU145 Cells. Evid. Based Complement. Altern. Med..

[145] Wu J.-W., Hsieh C.-L., Wang H.-Y., Chen H.-Y. (2009). Inhibitory Effects of Guava (*Psidium guajava* L.) Leaf Extracts and Its Active Compounds on the Glycation Process of Protein. Food Chem..

[146] Zhang G., Tang L., Liu H., Liu D., Wang M., Cai J., Liu W., Nie W., Zhang Y., Yu X. (2021). *Psidium guajava* Flavonoids Prevent NLRP3 Inflammasome Activation and Alleviate the Pancreatic Fibrosis in a Chronic Pancreatitis Mouse Model. Am. J. Chin. Med..

[147] Zioud F., Marzaioli V., El-Benna J., Bachoual R. (2019). *Punica granatum* and Citrillus Colocynthis Aqueous Extracts Protect Mice from LPS-Induced Lung Inflammation and Inhibit Metalloproteinases-2 and -9. Indian J. Pharm. Educ. Res..

[148] Kumagai Y., Nakatani S., Onodera H., Nagatomo A., Nishida N., Matsuura Y., Kobata K., Wada M. (2015). Anti-Glycation Effects of Pomegranate (*Punica granatum* L.) Fruit Extract and Its Components in Vivo and in Vitro. J. Agric. Food Chem..

[149] Aharoni S., Lati Y., Aviram M., Fuhrman B. (2015). Pomegranate Juice Polyphenols Induce a Phenotypic Switch in Macrophage Polarization Favoring a M2 Anti-Inflammatory State. BioFactors.

[150] Li X., Liu L., Pischetsrieder M. (2017). Pomegranate (*Punica granatum* L.) Wine Polyphenols Affect Nrf2 Activation and Antioxidant Enzyme Expression in Human Neuroblastoma Cells (SH-SY5Y). J. Funct. Foods.

[151] Kim H.Y., Kim K. (2003). Protein Glycation Inhibitory and Antioxidative Activities of Some Plant Extracts in Vitro. J. Agric. Food Chem..

[152] Lin G., Li N., Li D., Chen L., Deng H., Wang S., Tang J., Ouyang W. (2023). Carnosic Acid Inhibits NLRP3 Inflammasome Activation by Targeting Both Priming and Assembly Steps. Int. Immunopharmacol..

[153] Martin R., Pierrard C., Lejeune F., Hilaire P., Breton L., Bernerd F. (2008). Photoprotective Effect of a Water-Soluble Extract of Rosmarinus Officinalis L. against UV-Induced Matrix Metalloproteinase-1 in Human Dermal Fibroblasts and Reconstructed Skin. Eur. J. Dermatol..

[154] Yan M., Vemu B., Veenstra J., Petiwala S.M., Johnson J.J. (2018). Carnosol, a Dietary Diterpene from Rosemary (Rosmarinus Officinalis) Activates Nrf2 Leading to Sestrin 2 Induction in Colon Cells. Integr. Mol. Med..

[155] Bao B., Chen Y.-G., Zhang L., Na Xu Y.L., Wang X., Liu J., Qu W. (2013). Momordica Charantia (Bitter Melon) Reduces Obesity-Associated Macrophage and Mast Cell Infiltration as Well as Inflammatory Cytokine Expression in Adipose Tissues. PLoS ONE.

[156] Ashrafizadeh M., Ahmadi Z., Mohammadinejad R., Farkhondeh T., Samarghandian S. (2020). Curcumin Activates the Nrf2 Pathway and Induces Cellular Protection Against Oxidative Injury. Curr. Mol. Med..

[157] Guimarães I., Baptista-Silva S., Pintado M., Oliveira A.L. (2021). Polyphenols: A Promising Avenue in Therapeutic Solutions for Wound Care. Appl. Sci..

[158] Kaparekar P.S., Anandasadagopan S.K. (2022). The Potential Role of Bioactive Plant-Based Polyphenolic Compounds and Their Delivery Systems—As a Promising Opportunity for a New Therapeutic Solution for Acute and Chronic Wound Healing. Curr. Pharmacol. Rep..

[159] Johnson J.B., Broszczak D.A., Mani J.S., Anesi J., Naiker M. (2021). A Cut above the Rest: Oxidative Stress in Chronic Wounds and the Potential Role of Polyphenols as Therapeutics. J. Pharm. Pharmacol..

[160] Zulkefli N., Che Zahari C.N.M., Sayuti N.H., Kamarudin A.A., Saad N., Hamezah H.S., Bunawan H., Baharum S.N., Mediani A., Ahmed Q.U. (2023). Flavonoids as Potential Wound-Healing Molecules: Emphasis on Pathways Perspective. Int. J. Mol. Sci..

[161] Wang W., Sun C., Mao L., Ma P., Liu F., Yang J., Gao Y. (2016). The Biological Activities, Chemical Stability, Metabolism and Delivery Systems of Quercetin: A Review. Trends Food Sci. Technol..

[162] Fu J., Huang J., Lin M., Xie T., You T. (2020). Quercetin Promotes Diabetic Wound Healing via Switching Macrophages From M1 to M2 Polarization. J. Surg. Res..

[163] Ganesan S., Faris A.N., Comstock A.T., Chattoraj S.S., Chattoraj A., Burgess J.R., Curtis J.L., Martinez F.J., Zick S., Hershenson M.B. (2010). Quercetin Prevents Progression of Disease in Elastase/LPS-Exposed Mice by Negatively Regulating MMP Expression. Respir. Res..

[164] Bhuiyan M.N.I., Mitsuhashi S., Sigetomi K., Ubukata M. (2017). Quercetin Inhibits Advanced Glycation End Product Formation via Chelating Metal Ions, Trapping Methylglyoxal, and Trapping Reactive Oxygen Species. Biosci. Biotechnol. Biochem..

[165] Li R., Chen L., Yao G.-M., Yan H.-L., Wang L. (2021). Effects of Quercetin on Diabetic Retinopathy and Its Association with NLRP3 Inflammasome and Autophagy. Int. J. Ophthalmol..

[166] Yuan K., Zhu Q., Lu Q., Jiang H., Zhu M., Li X., Huang G., Xu A. (2020). Quercetin Alleviates Rheumatoid Arthritis by Inhibiting Neutrophil Inflammatory Activities. J. Nutr. Biochem..

[167] Lee D.E., Chung M.-Y., Lim T.G., Huh W.B., Lee H.J., Lee K.W. (2013). Quercetin Suppresses Intracellular ROS Formation, MMP Activation, and Cell Motility in Human Fibrosarcoma Cells. J. Food Sci..

[168] Kimura S., Warabi E., Yanagawa T., Ma D., Itoh K., Ishii Y., Kawachi Y., Ishii T. (2009). Essential Role of Nrf2 in Keratinocyte Protection from UVA by Quercetin. Biochem. Biophys. Res. Commun..

[169] Kim M.-R., Lee J.Y., Lee H.-H., Aryal D.K., Kim Y.G., Kim S.K., Woo E.-R., Kang K.W. (2006). Antioxidative Effects of Quercetin-Glycosides Isolated from the Flower Buds of *Tussilago Farfara* L.. Food Chem. Toxicol..

[170] Zheng Y.-Z., Deng G., Liang Q., Chen D.-F., Guo R., Lai R.-C. (2017). Antioxidant Activity of Quercetin and Its Glucosides from Propolis: A Theoretical Study. Sci. Rep..

[171] Calderon-Montano J.M., Burgos-Moron E., Perez-Guerrero C., Lopez-Lazaro M. (2011). A Review on the Dietary Flavonoid Kaempferol. Mini Rev. Med. Chem..

[172] Özay Y., Güzel S., Yumrutaş Ö., Pehlivanoğlu B., Erdoğdu İ.H., Yildirim Z., Türk B.A., Darcan S. (2019). Wound Healing Effect of Kaempferol in Diabetic and Nondiabetic Rats. J. Surg. Res..

[173] Ronsisvalle S., Panarello F., Longhitano G., Siciliano E.A., Montenegro L., Panico A. (2020). Natural Flavones and Flavonols: Relationships among Antioxidant Activity, Glycation, and Metalloproteinase Inhibition. Cosmetics.

[174] Zhao J., Ling L., Zhu W., Ying T., Yu T., Sun M., Zhu X., Du Y., Zhang L. (2023). M1/M2 Re-Polarization of Kaempferol Biomimetic NPs in Anti-Inflammatory Therapy of Atherosclerosis. J. Controlled Release.

[175] Lin C., Wu F., Zheng T., Wang X., Chen Y., Wu X. (2019). Kaempferol Attenuates Retinal Ganglion Cell Death by Suppressing NLRP1/NLRP3 Inflammasomes and Caspase-8 via JNK and NF-ΚB Pathways in Acute Glaucoma. Eye.

[176] Zeng J., Xu H., Fan P.-Z., Xie J., He J., Yu J., Gu X., Zhang C.-J. (2020). Kaempferol Blocks Neutrophil Extracellular Traps Formation and Reduces Tumour Metastasis by Inhibiting ROS-PAD4 Pathway. J. Cell. Mol. Med..

[177] Yao H., Sun J., Wei J., Zhang X., Chen B., Lin Y. (2020). Kaempferol Protects Blood Vessels From Damage Induced by Oxidative Stress and Inflammation in Association With the Nrf2/HO-1 Signaling Pathway. Front. Pharmacol..

[178] Imran M., Rauf A., Abu-Izneid T., Nadeem M., Shariati M.A., Khan I.A., Imran A., Orhan I.E., Rizwan M., Atif M. (2019). Luteolin, a Flavonoid, as an Anticancer Agent: A Review. Biomed. Pharmacother..

[179] Chen L.-Y., Cheng H.-L., Kuan Y.-H., Liang T.-J., Chao Y.-Y., Lin H.-C. (2021). Therapeutic Potential of Luteolin on Impaired Wound Healing in Streptozotocin-Induced Rats. Biomedicines.

[180] Saragusti A.C., Ortega M.G., Cabrera J.L., Estrin D.A., Marti M.A., Chiabrando G.A. (2010). Inhibitory Effect of Quercetin on Matrix Metalloproteinase 9 Activity Molecular Mechanism and Structure–Activity Relationship of the Flavonoid–Enzyme Interaction. Eur. J. Pharmacol..

[181] Pandurangan A., Dharmalingam P., Sadagopan S., Ganapasam S. (2014). Luteolin Inhibits Matrix Metalloproteinase 9 and 2 in Azoxymethane-Induced Colon Carcinogenesis. Hum. Exp. Toxicol..

[182] Sarmah S., Das S., Roy A.S. (2020). Protective Actions of Bioactive Flavonoids Chrysin and Luteolin on the Glyoxal Induced Formation of Advanced Glycation End Products and Aggregation of Human Serum Albumin: In Vitro and Molecular Docking Analysis. Int. J. Biol. Macromol..

[183] Wang S., Cao M., Xu S., Shi J., Mao X., Yao X., Liu C. (2020). Luteolin Alters Macrophage Polarization to Inhibit Inflammation. Inflammation.

[184] Lee M.N., Lee Y., Wu D., Pae M. (2021). Luteolin Inhibits NLRP3 Inflammasome Activation via Blocking ASC Oligomerization. J. Nutr. Biochem..

[185] Jablonska E., Garley M., Surazynski A., Grubczak K., Iwaniuk A., Borys J., Moniuszko M., Ratajczak-Wrona W. (2020). Neutrophil Extracellular Traps (NETs) Formation Induced by TGF-β in Oral Lichen Planus—Possible Implications for the Development of Oral Cancer. Immunobiology.

[186] Zhang B.-C., Li Z., Xu W., Xiang C.-H., Ma Y.-F. (2018). Luteolin Alleviates NLRP3 Inflammasome Activation and Directs Macrophage Polarization in Lipopolysaccharide-Stimulated RAW264.7 Cells. Am. J. Transl. Res..

[187] Cai Q., Rahn R.O., Zhang R. (1997). Dietary Flavonoids, Quercetin, Luteolin and Genistein, Reduce Oxidative DNA Damage and Lipid Peroxidation and Quench Free Radicals. Cancer Lett..

[188] Lopez-Lazaro M. (2009). Distribution and Biological Activities of the Flavonoid Luteolin. Mini Rev. Med. Chem..

[189] Lestari M.L.A.D., Indrayanto G. (2014). Chapter Three—Curcumin. Profiles Drug Subst. Excip. Relat. Methodol..

[190] Maheshwari R.K., Singh A.K., Gaddipati J., Srimal R.C. (2006). Multiple Biological Activities of Curcumin: A Short Review. Life Sci..

[191] Kant V., Gopal A., Kumar D., Pathak N.N., Ram M., Jangir B.L., Tandan S.K., Kumar D. (2015). Curcumin-Induced Angiogenesis Hastens Wound Healing in Diabetic Rats. J. Surg. Res..

[192] Kumar D., Kumar M., Saravanan C., Singh S.K. (2012). Curcumin: A Potential Candidate for Matrix Metalloproteinase Inhibitors. Expert Opin. Ther. Targets.

[193] Alizadeh M., Kheirouri S. (2019). Curcumin against Advanced Glycation End Products (AGEs) and AGEs-Induced Detrimental Agents. Crit. Rev. Food Sci. Nutr..

[194] Li M., Liu Z., Zhang Z., Ma L. (2006). Inhibitory Effects of Curcumin Derivatives on Nonenzymatic Glucosylation in Vitro. Front. Chem. China.

[195] Liu W., Ma H., DaSilva N.A., Rose K.N., Johnson S.L., Zhang L., Wan C., Dain J.A., Seeram N.P. (2016). Development of a Neuroprotective Potential Algorithm for Medicinal Plants. Neurochem. Int..

[196] Hu T.-Y., Liu C.-L., Chyau C.-C., Hu M.-L. (2012). Trapping of Methylglyoxal by Curcumin in Cell-Free Systems and in Human Umbilical Vein Endothelial Cells. J. Agric. Food Chem..

[197] Li B., Hu Y., Zhao Y., Cheng M., Qin H., Cheng T., Wang Q., Peng X., Zhang X. (2017). Curcumin Attenuates Titanium Particle-Induced Inflammation by Regulating Macrophage Polarization In Vitro and In Vivo. Front. Immunol..

[198] Momtazi-Borojeni A.A., Abdollahi E., Nikfar B., Chaichian S., Ekhlasi-Hundrieser M. (2019). Curcumin as a Potential Modulator of M1 and M2 Macrophages: New Insights in Atherosclerosis Therapy. Heart Fail. Rev..

[199] Gong Z., Zhou J., Li H., Gao Y., Xu C., Zhao S., Chen Y., Cai W., Wu J. (2015). Curcumin Suppresses NLRP3 Inflammasome Activation and Protects against LPS-Induced Septic Shock. Mol. Nutr. Food Res..

[200] Zhu C., Shi S., Jiang P., Huang X., Zhao J., Jin Y., Shen Y., Zhou X., Liu H., Cai J. (2023). Curcumin Alleviates Hepatic Ischemia-Reperfusion Injury by Inhibiting Neutrophil Extracellular Traps Formation. J. Investig. Surg..

[201] Ye S., Li S., Ma Y., Hu D., Xiao F. (2021). Curcumin Hinders PBDE-47-Induced Neutrophil Extracellular Traps Release via Nrf2-Associated ROS Inhibition. Ecotoxicol. Environ. Saf..

[202] Panchatcharam M., Miriyala S., Gayathri V.S., Suguna L. (2006). Curcumin Improves Wound Healing by Modulating Collagen and Decreasing Reactive Oxygen Species. Mol. Cell. Biochem..

[203] Tanaka T., Nonaka G.-I., Nishioka I. (1986). Tannins and Related Compounds. XL.: Revision of the Structures of Punicalin and Punicalagin, and Isolation and Characterization of 2-O-Galloylpunicalin from the Bark of *Punica granatum* L.. Chem. Pharm. Bull..

[204] Rozadi N., Oktavia S., Fauziah F. (2022). Pharmacological Activities of Punicalagin: A Review. J. Drug Deliv. Ther..

[205] Kumar A., Mishra R., Singh V., Mazumder A., Mazumder R., Kumar A. (2022). Wound Healing Activity of Punicalin and Punicalagin Isolated from *Punica granatum* L.. Rasayan J. Chem..

[206] Tang J., Li B., Hong S., Liu C., Min J., Hu M., Li Y., Liu Y., Hong L. (2017). Punicalagin Suppresses the Proliferation and Invasion of Cervical Cancer Cells through Inhibition of the β-Catenin Pathway. Mol. Med. Rep..

[207] Liu W., Ma H., Frost L., Yuan T., Dain J.A., Seeram N.P. (2014). Pomegranate Phenolics Inhibit Formation of Advanced Glycation Endproducts by Scavenging Reactive Carbonyl Species. Food Funct..

[208] Ge G., Bai J., Wang Q., Liang X., Tao H., Chen H., Wei M., Niu J., Yang H., Xu Y. (2022). Punicalagin Ameliorates Collagen-Induced Arthritis by Downregulating M1 Macrophage and Pyroptosis via NF-ΚB Signaling Pathway. Sci. China Life Sci..

[209] Lo J., Liu C.-C., Li Y.-S., Lee P.-Y., Liu P.-L., Wu P.-C., Lin T.-C., Chen C.-S., Chiu C.-C., Lai Y.-H. (2022). Punicalagin Attenuates LPS-Induced Inflammation and ROS Production in Microglia by Inhibiting the MAPK/NF-ΚB Signaling Pathway and NLRP3 Inflammasome Activation. J. Inflamm. Res..

[210] Sun Y., Tao X., Men X., Xu Z., Wang T. (2017). In Vitro and in Vivo Antioxidant Activities of Three Major Polyphenolic Compounds in Pomegranate Peel: Ellagic Acid, Punicalin, and Punicalagin. J. Integr. Agric..

[211] Aloqbi A., Omar U., Yousr M., Grace M., Lila M.A., Howell N. (2016). Antioxidant Activity of Pomegranate Juice and Punicalagin. Nat. Sci..

[212] Xu X., Li H., Hou X., Li D., He S., Wan C., Yin P., Liu M., Liu F., Xu J. (2015). Punicalagin Induces Nrf2/HO-1 Expression via Upregulation of PI3K/AKT Pathway and Inhibits LPS-Induced Oxidative Stress in RAW264.7 Macrophages. Mediators Inflamm..

[213] Zhao H., Huang J., Li Y., Lv X., Zhou H., Wang H., Xu Y., Wang C., Wang J., Liu Z. (2020). ROS-Scavenging Hydrogel to Promote Healing of Bacteria Infected Diabetic Wounds. Biomaterials.

[214] Al-Irayfawee N., Al-Biatey D., Hussein Z. (2019). Effectiveness of *Punica granatum* and Propolis: A New Dressing Method in Management of Diabetic Foot Ulceration. Indian J. Forensic Med. Toxicol..

[215] Mokhtari M., Razzaghi R., Momen-Heravi M. (2021). The Effects of Curcumin Intake on Wound Healing and Metabolic Status in Patients with Diabetic Foot Ulcer: A Randomized, Double-Blind, Placebo-Controlled Trial. Phytother. Res..

[216] Viswanathan V., Kesavan R., Kavitha K.V., Kumpatla S. (2011). A Pilot Study on the Effects of a Polyherbal Formulation Cream on Diabetic Foot Ulcers. Indian J. Med. Res..

[217] Carvalho M.T.B., Araújo-Filho H.G., Barreto A.S., Quintans-Júnior L.J., Quintans J.S.S., Barreto R.S.S. (2021). Wound Healing Properties of Flavonoids: A Systematic Review Highlighting the Mechanisms of Action. Phytomedicine.

[218] Lodhi S., Singhai A.K. (2013). Wound Healing Effect of Flavonoid Rich Fraction and Luteolin Isolated from Martynia Annua Linn. on Streptozotocin Induced Diabetic Rats. Asian Pac. J. Trop. Med..

[219] Chen L.-Y., Huang C.-N., Liao C.-K., Chang H.-M., Kuan Y.-H., Tseng T.-J., Yen K.-J., Yang K.-L., Lin H.-C. (2020). Effects of Rutin on Wound Healing in Hyperglycemic Rats. Antioxidants.

[220] Özay Y., Güzel S., Erdoğdu İ.H., Yıldırım Z., Pehlivanoğlu B., Aydın Türk B., Darcan S. (2018). Evaluation of the Wound Healing Properties of Luteolin Ointments on Excision and Incision Wound Models in Diabetic and Non-Diabetic Rats. Rec. Nat. Prod..

[221] Williams R.J., Spencer J.P.E., Rice-Evans C. (2004). Flavonoids: Antioxidants or Signalling Molecules?. Free Radic. Biol. Med..

[222] Burda S., Oleszek W. (2001). Antioxidant and Antiradical Activities of Flavonoids. J. Agric. Food Chem..

[223] Singh M.P., Gupta A., Sisodia S.S. (2020). Wound Healing Activity of Terminalia Bellerica Roxb. and Gallic Acid in Experimentally Induced Diabetic Animals. J. Complement. Integr. Med..

[224] Mo J., Panichayupakaranant P., Kaewnopparat N., Nitiruangjaras A., Reanmongkol W. (2014). Wound Healing Activities of Standardized Pomegranate Rind Extract and Its Major Antioxidant Ellagic Acid in Rat Dermal Wounds. J. Nat. Med..

[225] Chen Y.-J., Chang L.-S. (2012). Gallic Acid Downregulates Matrix Metalloproteinase-2 (MMP-2) and MMP-9 in Human Leukemia Cells with Expressed Bcr/Abl. Mol. Nutr. Food Res..

[226] Huang S.-T., Wang C.-Y., Yang R.-C., Wu H.-T., Yang S.-H., Cheng Y.-C., Pang J.-H.S. (2011). Ellagic Acid, the Active Compound of *Phyllanthus Urinaria*, Exerts *In Vivo* Anti-Angiogenic Effect and Inhibits MMP-2 Activity. Evid. Based Complement. Altern. Med..

[227] Mao H., Guan N., Gao X., Zhang W., Xue G., Wang L. (2022). Gallic Acid Inhibits M1 Macrophage Polarization via Adenosine 5′-Monophosphate-Activated Protein Kinase/Signal Transducers and Activators of Transcription 1 Pathway. Indian J. Pharm. Sci..

[228] Koleckar V., Kubikova K., Rehakova Z., Kuca K., Jun D., Jahodar L., Opletal L. (2008). Condensed and Hydrolysable Tannins as Antioxidants Influencing the Health. Mini Rev. Med. Chem..

[229] Zhou D., Yang Q., Tian T., Chang Y., Li Y., Duan L.-R., Li H., Wang S.-W. (2020). Gastroprotective Effect of Gallic Acid against Ethanol-Induced Gastric Ulcer in Rats: Involvement of the Nrf2/HO-1 Signaling and Anti-Apoptosis Role. Biomed. Pharmacother..

[230] Altamimi J.Z., AlFaris N.A., Alshammari G.M., Alagal R.I., Aljabryn D.H., Aldera H., Alrfaei B.M., Alkhateeb M.A., Yahya M.A. (2021). Ellagic Acid Protects against Diabetic Nephropathy in Rats by Regulating the Transcription and Activity of Nrf2. J. Funct. Foods.

[231] Tsai F.-S., Lin L.-W., Wu C.-R. (2016). Lupeol and Its Role in Chronic Diseases. Drug Discov. Mother Nat..

[232] Zhu Y., Li X., Chen J., Chen T., Shi Z., Lei M., Zhang Y., Bai P., Li Y., Fei X. (2016). The Pentacyclic Triterpene Lupeol Switches M1 Macrophages to M2 and Ameliorates Experimental Inflammatory Bowel Disease. Int. Immunopharmacol..

[233] Oliveira-Junior M.S., Pereira E.P., de Amorim V.C.M., Reis L.T.C., do Nascimento R.P., da Silva V.D.A., Costa S.L. (2019). Lupeol Inhibits LPS-Induced Neuroinflammation in Cerebellar Cultures and Induces Neuroprotection Associated to the Modulation of Astrocyte Response and Expression of Neurotrophic and Inflammatory Factors. Int. Immunopharmacol..

[234] Zhang Z., Xu C., Hao J., Zhang M., Wang Z., Yin T., Lin K., Liu W., Jiang Q., Li Z. (2020). Beneficial Consequences of Lupeol on Middle Cerebral Artery-Induced Cerebral Ischemia in the Rat Involves Nrf2 and P38 MAPK Modulation. Metab. Brain Dis..

[235] Beserra F.P., Vieira A.J., Gushiken L.F.S., de Souza E.O., Hussni M.F., Hussni C.A., Nóbrega R.H., Martinez E.R.M., Jackson C.J., de Azevedo Maia G.L. (2019). Lupeol, a Dietary Triterpene, Enhances Wound Healing in Streptozotocin-Induced Hyperglycemic Rats with Modulatory Effects on Inflammation, Oxidative Stress, and Angiogenesis. Oxid. Med. Cell. Longev..

